# The potential of hypoxia markers as target for breast molecular imaging – a systematic review and meta-analysis of human marker expression

**DOI:** 10.1186/1471-2407-13-538

**Published:** 2013-11-10

**Authors:** Arthur Adams, Aram SA van Brussel, Jeroen F Vermeulen, Willem PThM Mali, Elsken van der Wall, Paul J van Diest, Sjoerd G Elias

**Affiliations:** 1 Department of Radiology, University Medical Center Utrecht, Utrecht, The Netherlands; 2 Department of Pathology, University Medical Center Utrecht, Utrecht, The Netherlands; 3 Division of Internal Medicine and Dermatology, University Medical Center Utrecht, Utrecht, The Netherlands; 4 Julius Center of Health Sciences and Primary Care, University Medical Center Utrecht, Utrecht, The Netherlands

**Keywords:** Breast cancer, Carbonic anhydrase-IX, CAIX, Glucose transporter-1, GLUT1, C-X-C chemokine receptor type-4, CXCR4, Insulin-like growth factor-1 receptor, IGF1R, Expression prevalence, Systematic review, Meta-analysis, Molecular imaging, Immunohistochemistry, Carcinoma *in situ*, Benign breast disease, Normal breast tissue

## Abstract

**Background:**

Molecular imaging of breast cancer is a promising emerging technology, potentially able to improve clinical care. Valid imaging targets for molecular imaging tracer development are membrane-bound hypoxia-related proteins, expressed when tumor growth outpaces neo-angiogenesis. We performed a systematic literature review and meta-analysis of such hypoxia marker expression rates in human breast cancer to evaluate their potential as clinically relevant molecular imaging targets.

**Methods:**

We searched MEDLINE and EMBASE for articles describing membrane-bound proteins that are related to hypoxia inducible factor 1α (HIF-1α), the key regulator of the hypoxia response. We extracted expression rates of carbonic anhydrase-IX (CAIX), glucose transporter-1 (GLUT1), C-X-C chemokine receptor type-4 (CXCR4), or insulin-like growth factor-1 receptor (IGF1R) in human breast disease, evaluated by immunohistochemistry. We pooled study results using random-effects models and applied meta-regression to identify associations with clinicopathological variables.

**Results:**

Of 1,705 identified articles, 117 matched our selection criteria, totaling 30,216 immunohistochemistry results. We found substantial between-study variability in expression rates. Invasive cancer showed pooled expression rates of 35% for CAIX (95% confidence interval (CI): 26-46%), 51% for GLUT1 (CI: 40-61%), 46% for CXCR4 (CI: 33-59%), and 46% for IGF1R (CI: 35-70%). Expression rates increased with tumor grade for GLUT1, CAIX, and CXCR4 (all p < 0.001), but decreased for IGF1R (p < 0.001). GLUT1 showed the highest expression rate in grade III cancers with 58% (45-69%). CXCR4 showed the highest expression rate in small T1 tumors with 48% (CI: 28-69%), but associations with size were only significant for CAIX (p < 0.001; positive association) and IGF1R (p = 0.047; negative association). Although based on few studies, CAIX, GLUT1, and CXCR4 showed profound lower expression rates in normal breast tissue and benign breast disease (p < 0.001), and high rates in carcinoma *in situ*. Invasive lobular carcinoma consistently showed lower expression rates (p < 0.001).

**Conclusions:**

Our results support the potential of hypoxia-related markers as breast cancer molecular imaging targets. Although specificity is promising, combining targets would be necessary for optimal sensitivity. These data could help guide the choice of imaging targets for tracer development depending on the envisioned clinical application.

## Background

In the past decades, conventional breast imaging modalities such as (digital) mammography, breast ultrasound, and more recently dynamic contrast enhanced magnetic resonance imaging (DCE-MRI), have improved detection, characterization, and management of breast cancer. Although these imaging modalities are valuable in clinical practice, novel imaging strategies such as molecular imaging promise additional advantages. With molecular imaging techniques, breast cancer could be detected even before anatomical changes occur that are required for visualization with currently used imaging modalities, making it valuable for early detection or screening. For diagnostic purposes, more informative characterization of breast cancer could result in less unnecessary biopsies. Furthermore, improved imaging of the extent of disease could lead to better preoperative planning and to per-operative guidance, increasing the primary surgery success rate. Molecular imaging could also be applied to demonstrate the presence of appropriate molecular targets in the primary tumor, lymph node and distant metastasis (*in vivo* receptor status determination), and could therefore be useful to tailor therapy to individual patients and to monitor therapy response [1-6]. Molecular imaging of tumor metabolism using ^18^F-fluorodeoxyglucose (^18^F-FDG) Positron Emission Tomography is currently common for imaging and staging of advanced breast cancer. However, it is of limited value in evaluation of early breast cancer because of limited spatial resolution, non-visibility of tumors with low ^18^F-FDG avidity, and low specificity [7].

Imaging of tumor hypoxia could be a feasible alternative strategy for molecular imaging of breast cancer. Hypoxia is a frequent phenomenon in solid tumors that arises due to limited perfusion [8,9], and might therefore be more specific than ^18^F-FDG imaging. Direct imaging of tumor hypoxia using oxygen mimetics (e.g. with radiolabelled 2-nitroimidazole derivatives (^18^F-FMISO, ^18^F-FAZA, ^18^F-EF5) and other molecules such as Cu-ATSM) has been investigated in several clinical studies [10]. However, the biodistribution properties of these molecules result in images with low contrast.

Molecular imaging using (monoclonal) antibodies or antibody fragments (e.g. single chain variable fragments (scFv), antibody-binding fragments (Fab), variable domains of the heavy chain of heavy chain-only antibodies (VHH) or affibodies) that have high affinity for markers that are expressed in breast cancer under hypoxic conditions could improve imaging contrast [11-13]. The molecules that are targeted with these antibodies or fragments should ideally be highly prevalent in (breast) cancer, and expression should preferably be already present at the initial stage of tumorigenesis. Expression of these molecules should be absent or low in non-affected tissue and benign breast disease for high specificity, although the relative importance of these properties depends on the envisioned clinical application. For screening purposes, specificity of the target of interest should be high and for application in a diagnostic setting, expression prevalence of the target in breast cancer should be sufficient. For intra-operative guidance, high expression prevalences are less important as pre-operative target selection is possible based on a diagnostic (core) biopsy. However, distribution of the target within the tumor should be homogenous when used for assessment of tumor margins. Furthermore, extracellular membrane bound molecules are most attractive, as these are more easily accessible for most antibodies or antibody fragments compared to intracellular molecules [14].

Hypoxic conditions result in focal expression of hypoxia inducible factor 1α (HIF-1α), the key regulator of the hypoxia response [8,15,16]. The downstream targets of HIF-1α, carbonic anhydrase IX (CAIX), glucose transporter 1 (GLUT1) and C-X-C chemokine receptor type 4 (CXCR4) [17-20], and insulin-like growth factor 1 receptor (IGF1R) that maintains the hypoxia response via HIF-1α stabilization [21-23], are expressed on the plasma membrane of breast cancer cells and are therefore potentially suitable candidates for molecular imaging of hypoxic tumors with antibodies or antibody fragments.

Despite the apparent potential of these hypoxia related proteins, expression patterns in human breast cancer, normal breast tissue and benign breast diseases, as well as expression in tumor margins and heterogeneity within tumors are not well established. To evaluate whether molecular imaging using these targets could be clinically relevant, we performed a systematic literature review and meta-analysis to quantify expression prevalences of these hypoxia markers in breast disease as assessed by immunohistochemistry (IHC), investigated relations with clinicopathological characteristics, and assessed the influence of specimen handling on these prevalences. These data could help guide the choice of relevant imaging targets for future tracer development towards clinical studies.

## Methods

### Literature search

We performed a systematic search in the databases of MEDLINE and EMBASE on August 21^st^, 2012. Search terms included synonyms for the targets of interest (CXCR4, GLUT1, CAIX, and IGF1R), combined with ‘breast’ and ‘mamm*’. The full search syntax can be found in Table 1. We applied no restrictions on publication date. The search in the database of EMBASE was limited to articles that were not indexed with a MEDLINE ID, and conference abstracts were excluded. Duplicate articles were manually removed from the search results.

**Table 1:** Search strategy used to identify publications of interest regarding prevalence of hypoxia proteins in benign and malignant breast tissue

**Target**	**Synonyms used**
CAIX	CAIX OR CA-IX OR “CA IX” OR CA9 OR CA-9 OR “CA 9” OR “carbonic anhydrase IX” OR “carbonic anhydrase 9”
GLUT1	GLUT1 OR GLUT-1 OR “glucose transporter 1”
CXCR4	CXCR4 OR CXCR-4 OR CXC-R4 OR “CXC chemokine receptor-4”
IGF1R	“insulin like growth factor 1 receptor” OR “insulin like growth factor I receptor” OR IGF1R OR IGF-1R OR IGFR OR IGF-IR OR IGF1-R

Search terms were combined with ‘breast’ and ‘mamm*’. For MEDLINE, ‘[tiab]’ was added to each search term, and for EMBASE, ‘ti;ab;’ was added to each search term.

### Article selection

**Figure 1:**
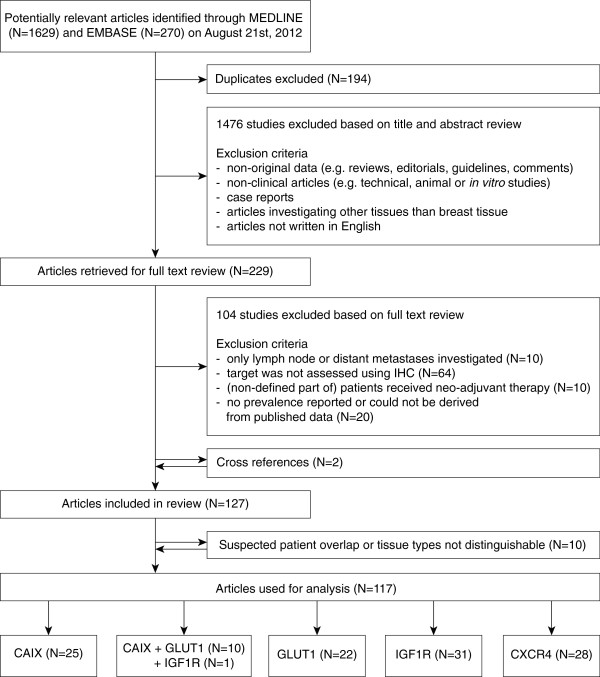
**Flowchart for selection of articles describing expression prevalences of the hypoxia markers CAIX, GLUT1, CXCR4, and IGF1R in breast cancer, normal tissue, benign breast disease, and carcinoma *in situ*, assessed by immunohistochemistry.**

Article eligibility was assessed by three reviewers (AA, AvB, JV) through independent screening of all titles and abstracts from the search result (triple read). We excluded articles based on predefined criteria, disagreements were resolved by discussion. An overview of the selection procedure is shown in Figure 1. Reasons for exclusion of articles based on title or abstract were: (1) non-original data (e.g. reviews, editorials, guidelines, and comments), (2) non-clinical articles (e.g. technical, animal, or *in vitro* studies), (3) case reports, (4) articles investigating other tissues than breast tissue, or (5) articles not written in the English language. The full texts of the remaining articles were screened for expression prevalence of the targets of interest. Studies were excluded if (1) only lymph node or distant metastases were investigated (N = 10), (2) the target of interest was assessed with another method than IHC (e.g. quantitative Polymerase Chain Reaction or Western Blot, N = 64), (3) all or a non-definable part of patients received neo-adjuvant therapy (which could profoundly alter biomarker status, N = 10), or (4) the prevalence of the target of interest was not reported and could not be derived from the published data (N = 20). All references of the remaining articles were reviewed to retrieve articles initially missed in the search syntax.

### Data extraction and statistical analysis

We extracted relevant information of each study (e.g. study and population characteristics, patient and tumor characteristics, and IHC methodology). Then, for each study and per target of interest, we annotated the number of lesions stated as target-positive and the total number of lesions, either directly or through recalculation based on the information stated in the article. Lesions of interest were invasive breast cancers, carcinoma *in situ*, benign breast lesions, or normal breast tissue. For invasive cancers, we grouped studies describing similar cut-off levels for marker positivity. When a study described multiple cut-off levels, the level corresponding to the most used cut-off among other included studies was used, as established after collecting all data. If patient data was used in more than one article (i.e. when articles referred to the same study, or assessed a comparable number of patients from the same hospital in a similar inclusion period to evaluate the expression of the same hypoxia marker), then only the article with the largest number of patients was included in the review and meta-analysis. A subgroup was defined for studies investigating membranous staining patterns only. Also, in order to assess applicability of the targets for human molecular imaging studies, we identified articles using a stringent or high cut-off value and preferentially membranous staining localization, as these studies provide the best evidence for high expression levels of the target. Furthermore, subgroups were defined according to tumor size (based on the TNM staging system), histological grade, histological subtype, and specimen handling method (i.e. if full tissue sections or tissue microarrays (TMA) were investigated), when stated. To assess specificity of the investigated markers, studies were grouped according to tissue types other than invasive breast cancer (normal tissue, benign breast disease, carcinoma *in situ*).

Then, we pooled prevalence rates across studies using a random-effects model, allowing for between-study heterogeneity. We fitted a linear mixed model using the exact binomial approach with the restricted maximum likelihood method [24]. We tested for subgroup differences using meta-regression analysis with subgroup indicators as fixed effects and the individual studies as random effects in the models. Besides the pooled prevalence estimates, we report predictive intervals as suggested by Higgins et al. for the evaluation of between-study heterogeneity [25]. We evaluated presence of publication bias with funnel plots and statistically tested for funnel plot asymmetry using Egger’s test [26].

Analyses were performed with R (version 2.15.1, R Foundation for Statistical Computing, Vienna, Austria) [27] with the package ‘lme4’ [28] and ‘meta’ [29]. All statistical tests were two-sided and a p-value of 0.05 or less was considered statistically significant. Prevalence estimates are reported with corresponding 95% logit confidence intervals (CI).

## Results

The search yielded 1,629 articles in MEDLINE and 270 articles in EMBASE. After removal of 194 duplicates, 1,705 unique articles were left for evaluation. Of these, we excluded 1,476 articles based on title and abstract, and 104 articles based on full text screening (Figure 1). Reference cross-checking of the selected articles yielded two additional studies that were initially missed, as synonyms for breast were not included in the title or abstract [30,31]. Of the 127 selected articles (CAIX [9,32-71], GLUT1 [30,31,33,34,36,39,42,45],[46,49,53,62,65,67,69,72-91], CXCR4 [92-121] IGF1R [36,122-156]), we excluded ten articles from the analysis due to (suspected) overlap of study populations [38,43,61,62,94,109,123,139],[143,153], and one article [67] because we could not distinguish between carcinoma *in situ* and invasive breast cancer. Ten articles [33,34,39,42,45,46,49,53],[65,69] described both GLUT1 and CAIX expression, and one study [36] described IGF1R, CAIX, and GLUT1 expression. In three of these studies, co-expression patterns of CAIX and GLUT1 were investigated [42,45,69]. Study characteristics of all investigated studies are shown in Additional file 1: Table S1A, Additional file 2: Table S1B, Additional file 3: Table S1C, Additional file 4: Table S1D.

IHC methodology varied between the studies. For assessment of CAIX expression, three different antibodies were used, and in 11 studies (31%) only the manufacturer was stated. In articles describing GLUT1 prevalence, six different antibodies were used and in 23 studies (70%) only the manufacturer was stated. For CXCR4, eight antibodies were used and in seven studies (25%) the antibody data was not reported, and for IGF1R, 11 different antibodies were used, and five studies (16%) did not specify the clone used. In addition, 51 studies (44%) investigated TMAs to evaluate the expression of the target of interest. Only 32 studies (63%) using TMAs reported the number of cores, and 37 studies (73%) reported the diameter of the cores. In 43 of the studies (37%) no information was available on who assessed staining results, 18 studies (15%) reported evaluation by a single observer and in 56 studies (48%) by more than one observer. In 43 of the studies (37%), it was explicitly stated that evaluation was performed by one or more pathologists.

### CAIX

**Figure 2:**
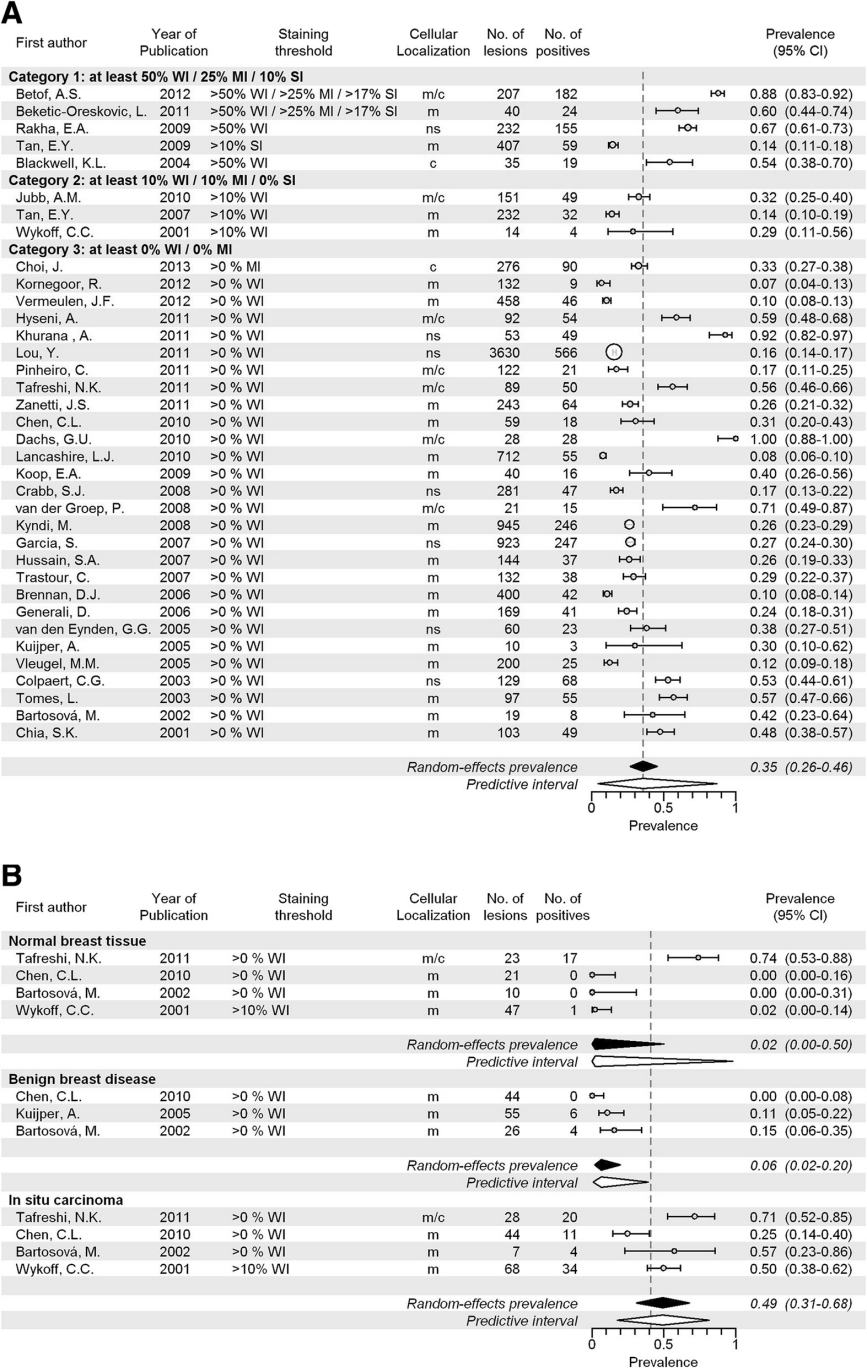
**Expression prevalence of CAIX. A** Systematic literature review of CAIX prevalence in breast cancer assessed by immunohistochemistry, according to reported staining threshold. Legend: Dashed gray reference line: overall random-effects prevalence estimate. Abbreviations: Staining threshold: weak intensity (WI), moderate intensity (MI), strong intensity (SI); Localization: cytoplasm (c), membrane (m); confidence interval (CI); not stated (NS). **B** Systematic literature review of CAIX prevalence in normal breast tissue, benign breast diseases and carcinoma in situ assessed by immunohistochemistry. Legend: Dashed line represents random effect summary prevalence estimate for invasive cancer within studies reporting also on normal, benign and/or precancerous breast tissue (4 studies). Abbreviations: Staining threshold: weak intensity (WI), moderate intensity (MI), strong intensity (SI); Localization: cytoplasm (c), membrane (m); confidence interval (CI); not stated (NS).

A total of 36 articles including 10,885 invasive cancers (range of 10 to 3,630 cancers per study) reported on CAIX expression, with prevalence estimates ranging from 7% to 92%. The overall pooled prevalence of CAIX was 35% (CI 26-46%; Figure 2A and Table 2). For studies investigating membranous staining patterns only, we found a pooled expression prevalence of 23% (CI 17-31%, 20 studies; Additional file 5: Figure S1A) and the studies providing best evidence for evaluation of molecular imaging targets showed a pooled prevalence of 38% (CI 17-65%, 6 studies; Additional file 6: Figure S1B). Expression prevalence of CAIX increased with histological grade (16% in grade II (p < 0.001) and 30% in grade III (p < 0.001) versus 4% in grade I; Additional file 7: Figure S1C), and tumor size (15% in T2 (p < 0.001) and 30% in T3 (p < 0.001) versus 12% in T1; Additional file 8: Figure S1D). Prevalence of CAIX was also higher in invasive ductal carcinoma (IDC) compared to invasive lobular carcinoma (ILC) (34% versus 1%, p = 0.001; Additional file 9: Figure S1E). CAIX expression was more often positive in studies investigating full sections compared to TMA (51% versus 24%, p = 0.002; Additional file 10: Figure S1F). In normal breast tissue, the pooled prevalence was 2% (CI 0-50%, p < 0.001; 4 studies). Pooled prevalence in benign lesions was 6% (CI 2-20%, p < 0.001; 3 studies), and in carcinoma *in situ* 49% (CI 31-68%, p = 0.025; 4 studies) (Figure 2B). Overall, between study-heterogeneity of studies investigating CAIX expression was large, but this decreased when confining analyses to membranous-only and best evidence studies (these study groups largely overlapped). Between-study variation was also lower within subgroups of tumor grade and tumor size.

**Table 2:** **Systematic review, meta-analysis and meta-regression results of hypoxia membrane protein expression in breast cancer, *in situ *** **carcinoma, benign breast disease, and normal breast tissue.**

	**CAIX**				**GLUT1**				**CXCR4**				**IGF1R**			
	**N**	**Prev.**	**(CI)**	p-value*	**N**	**Prev.**	**(CI)**	p-value*	**N**	**Prev.**	**(CI)**	p-value*	**N**	**Prev.**	**(CI)**	p-value*
***Invasive carcinoma***																
Overall	36	0.35	(0.26-0.46)	Ref	33	0.51	(0.40-0.61)	Ref	28	0.46	(0.33-0.59)	Ref	31	0.46	(0.35-0.70)	Ref
Membranous localization only	20	0.23	(0.17-0.31)	-	19	0.44	(0.37-0.52)	-	2	0.16	(0.08-0.31)	-	15	0.38	(0.27-0.50)	-
Best evidence studies	6	0.38	(0.17-0.65)	-	17	0.41	(0.35-0.48)	-	7	0.43	(0.25-0.63)	-	10	0.33	(0.22-0.46)	-
***Histological grade***	12				10				13				5			
I		0.04	(0.02-0.08)	Ref		0.24	(0.18-0.31)	Ref		0.26	(0.13-0.44)	Ref		0.57	(0.51-0.63)	Ref
II		0.16	(0.10-0.24)	<0.001		0.33	(0.20-0.50)	0.012		0.32	(0.17-0.52)	0.049		0.51	(0.49-0.54)	0.093
III		0.30	(0.22-0.39)	<0.001		0.58	(0.45-0.69)	<0.001		0.44	(0.26-0.63)	<0.001		0.41	(0.39-0.43)	<0.001
***Tumor size***	7				6				12				4			
T1		0.12	(0.11-0.14)	Ref		0.37	(0.31-0.42)	Ref		0.48	(0.28-0.69)	Ref		0.45	(0.39-0.51)	Ref
T2		0.15	(0.11-0.20)	<0.001		0.36	(0.29-0.43)	0.641		0.52	(0.28-0.74)	0.620		0.47	(0.44-0.49)	0.682
T3		0.30	(0.17-0.47)	<0.001		0.30	(0.14-0.53)	0.180		0.68	(0.53-0.80)	0.122		0.39	(0.32-0.47)	0.047
***Histological type***	13				14				10				6			
Invasive ductal carcinoma		0.34	(0.20-0.52)	Ref		0.48	(0.32-0.64)	Ref		0.46	(0.22-0.72)	Ref		0.42	(0.28-0.58)	Ref
Invasive lobular carcinoma		0.01	(0.00-0.05)	0.001		0.09	(0.01-0.40)	<0.001		0.35	(0.00-0.98)	0.001		0.25	(0.08-0.55)	<0.001
***Specimen handling***	36				32				28				31			
Full sections		0.51	(0.37-0.64)	Ref		0.61	(0.49-0.72)	Ref		0.39	(0.28-0.51)	Ref		0.34	(0.26-0.42)	Ref
Tissue microarray		0.24	(0.16-0.35)	0.002		0.30	(0.18-0.45)	0.003		0.61	(0.29-0.85)	0.173		0.57	(0.39-0.73)	0.032
***Other tissue types***	4				5				6				4			
Normal breast tissue		0.02	(0.00-0.50)	<0.001		0.03	(0.00-0.22)	<0.001		0.03	(0.01-0.07)	<0.001		0.74	(0.69-0.78)	0.109
Benign breast diseases		0.06	(0.02-0.20)	<0.001		0.04	(0.00-0.42)	<0.001		0.04	(0.00-0.80)	<0.001		0.73	(0.66-0.79)	0.137
Carcinoma *in situ*		0.49	(0.31-0.68)	0.025		0.52	(0.42-0.62)	0.680		0.71	(0.23-0.95)	<0.001		0.33	(0.18-0.53)	0.869

*p-values obtained using meta-regression (linear mixed model with subgroup indicators as fixed and the individual studies as random effects); ref: reference category for the meta-regression result; N: Maximum number of studies evaluated for pooled estimate or meta regression; prev.: expression prevalence of the investigated target.

### GLUT1

**Figure 3:**
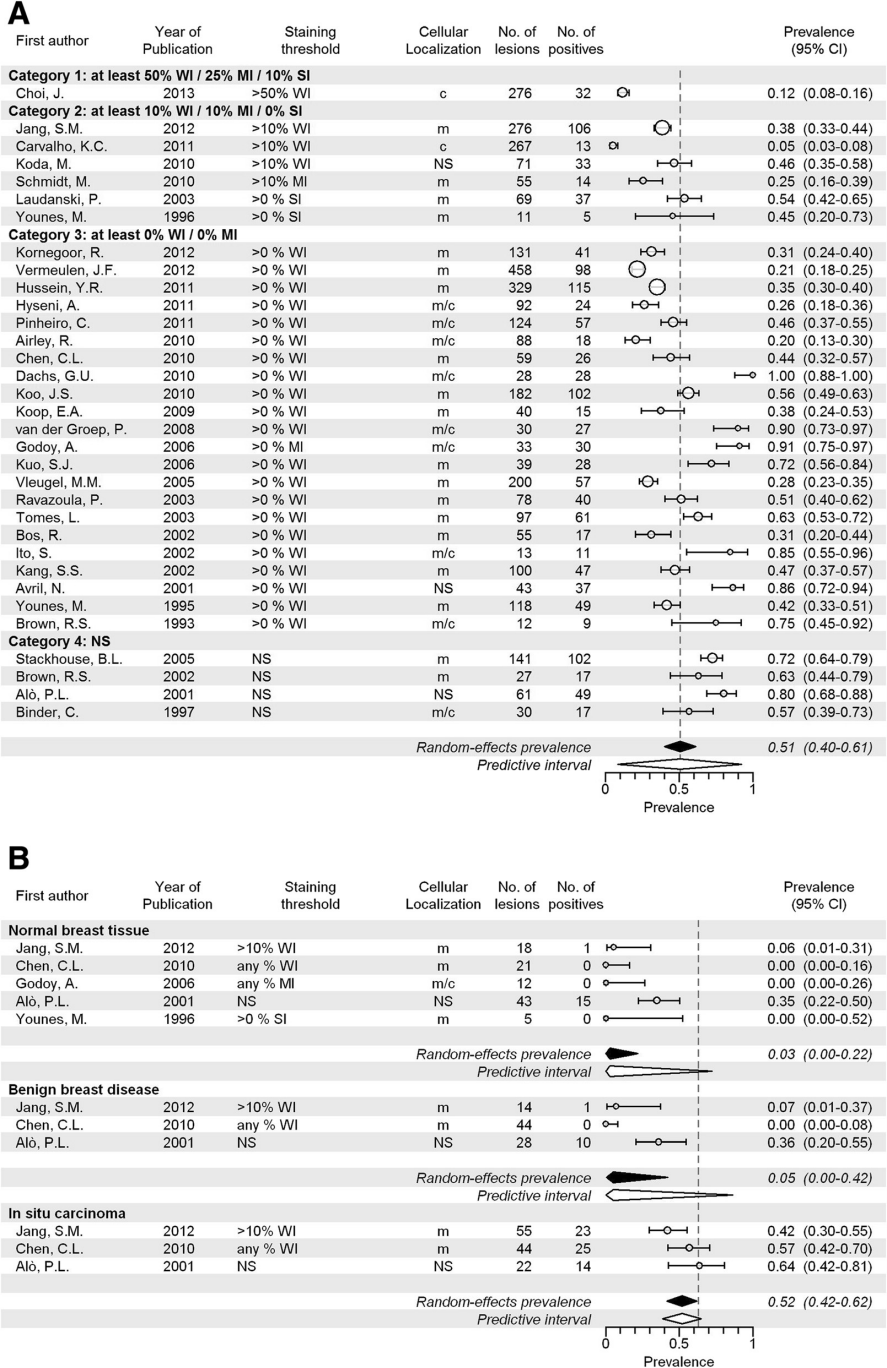
**Expression prevalence of GLUT1. A** Systematic literature review of GLUT1 prevalence in breast cancer assessed by immunohistochemistry, according to reported staining threshold. Legend: Dashed gray reference line: overall random-effects prevalence estimate. Abbreviations: Staining threshold: weak intensity (WI), moderate intensity (MI), strong intensity (SI); Localization: cytoplasm (c), membrane (m); confidence interval (CI); not stated (NS). **B** Systematic literature review of GLUT 1 prevalence in normal breast tissue, benign breast diseases and carcinoma in situ assessed by immunohistochemistry. Legend: Dashed line represents random effect summary prevalence estimate for invasive cancer within studies reporting also on normal, benign and/or precancerous breast tissue ( 5 studies). Abbreviations: Staining threshold: weak intensity (WI), moderate intensity (MI), strong intensity (SI); Localization: cytoplasm (c), membrane (m); confidence interval (CI); not stated (NS).

A total of 33 articles including 3,633 invasive cancers reported on GLUT1 expression, with a range of 11 to 458 cancers per study. The overall pooled prevalence of GLUT1 expression was 51% (CI 40-61%; Figure 3A and Table 2), but the reported prevalence varied substantially between studies (range 5% to 100%). For studies investigating membranous staining patterns only, the pooled prevalence was 44% (CI 37-52%, 19 studies; Additional file 11: Figure S2A) and when the studies providing best evidence for evaluation of molecular imaging targets were selected, this was 41% (CI 35-48%; 17 studies; Additional file 12: Figure S2B). GLUT1 prevalence was higher for grade III (58%, p < 0.001) and grade II tumors (33%, p = 0.012) compared to grade I tumors (24%; Additional file 13: Figure S2C), but there was no relation with tumor size (Additional file 14: Figure S2D). Furthermore, as for CAIX, expression prevalence in ILC was lower compared to IDC (9% versus 48%, p < 0.001; Additional file 15: Figure S2E). Studies investigating TMAs reported lower prevalence of GLUT1 expression compared to studies using full sections (30% versus 61%, p = 0.003, Additional file 16: Figure S2F). In normal breast tissue, the pooled expression prevalence was 3% (CI 0-22%, p < 0.001; 5 studies). Pooled prevalence in benign lesions was 5% (CI 0-42%, p < 0.001; 3 studies), and in carcinoma *in situ* 52% (CI 42-62%, p = 0.680; 3 studies) (Figure 3B). For GLUT1, the overall between-study variation was large as well, but substantially smaller for studies investigating membranous staining only and the best evidence studies (these study groups again largely overlapped). Furthermore, the between-study variation was markedly lower when taking tumor size into account, and somewhat lower within subgroups of grade. In the studies investigating co-expression patterns of GLUT1 and CAIX, concordant presence or absence of CAIX and GLUT1 was found in 78/118 (66%) [42], 45/59 (76%) [45], and 45/48 (94%) [69] of the cancers, respectively.

### CXCR4

**Figure 4:**
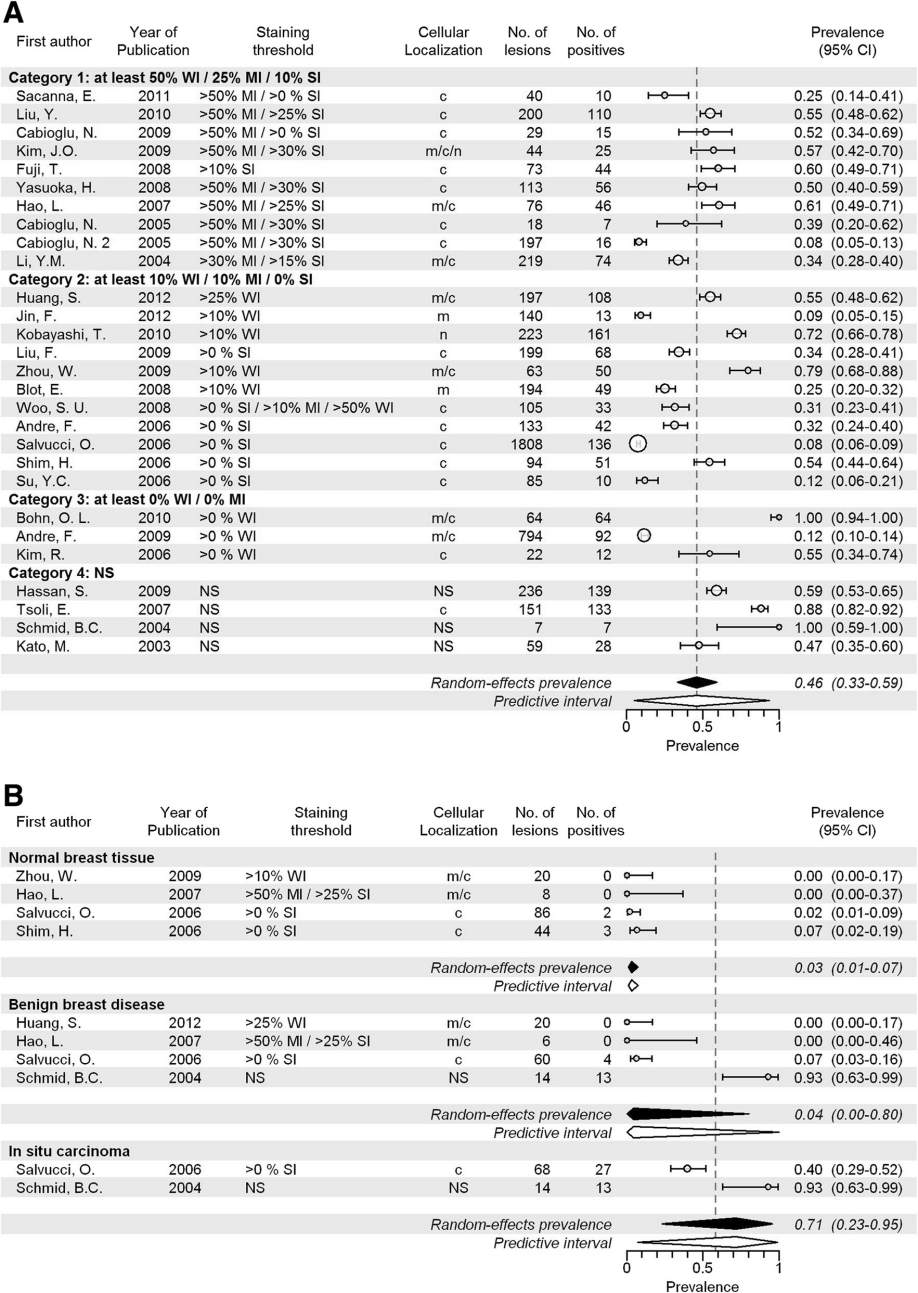
**Expression prevalence of CXCR4. A** Systematic literature review of CXCR4 prevalence in breast cancer assessed by immunohistochemistry, according to reported staining threshold. Legend: Dashed gray reference line: overall random-effects prevalence estimate. Abbreviations: Staining threshold: weak intensity (WI), moderate intensity (MI), strong intensity (SI); Localization: cytoplasm (c), membrane (m); confidence interval (CI); not stated (NS). **B** Systematic literature review of CXCR4 prevalence in normal breast tissue, benign breast diseases and carcinoma in situ assessed by immunohistochemistry. Legend: Dashed line represents random effect summary prevalence estimate for invasive cancer within studies reporting also on normal, benign and/or precancerous breast tissue (6 studies). Abbreviations: Staining threshold: weak intensity (WI), moderate intensity (MI), strong intensity (SI); Localization: cytoplasm (c), membrane (m); confidence interval (CI); not stated (NS).

A total of 28 articles including 5,583 invasive cancers reported on CXCR4 expression, with a range of 7 to 1,808 cancers per study. The pooled prevalence of CXCR4 expression was 46% (CI 33-59%; Figure 4A and Table 2), with a range between studies of 8% to 100%. For studies investigating membranous staining patterns only, the pooled prevalence was 16% (CI 8-31%; 2 studies; Additional file 17: Figure S3A) and when the studies providing best evidence for evaluation of molecular imaging targets were selected, this was 43% (CI 25-63%; 7 studies, Additional file 18: Figure S3B). CXCR4 prevalence increased with histological grade (32% in grade II (p = 0.049) and 44% in grade III (p < 0.001), compared to 26% in grade I; Additional file 19: Figure S3C), but no relation was found with tumor size (Additional file 20: Figure S3D). Furthermore, the prevalence of CXCR4 was higher in IDC than in ILC (46% versus 35%, p = 0.001; Additional file 21: Figure S3E). Expression prevalence was not related to slide construction method (Additional file 22: Figure S3F). In normal breast tissue, the pooled expression prevalence was 3% (CI 1-7%, p < 0.001; 4 studies). Pooled prevalence in benign lesions was 4% (CI 0-80%, p < 0.001; 4 studies), and in carcinoma *in situ* 71% (CI 23-95%, p < 0.001; 2 studies) (Figure 4B). Between-study heterogeneity of studies investigating CXCR4 expression was large, both overall and within all subgroups (except for the two studies investigating membranous staining).

### IGF1R

**Figure 5:**
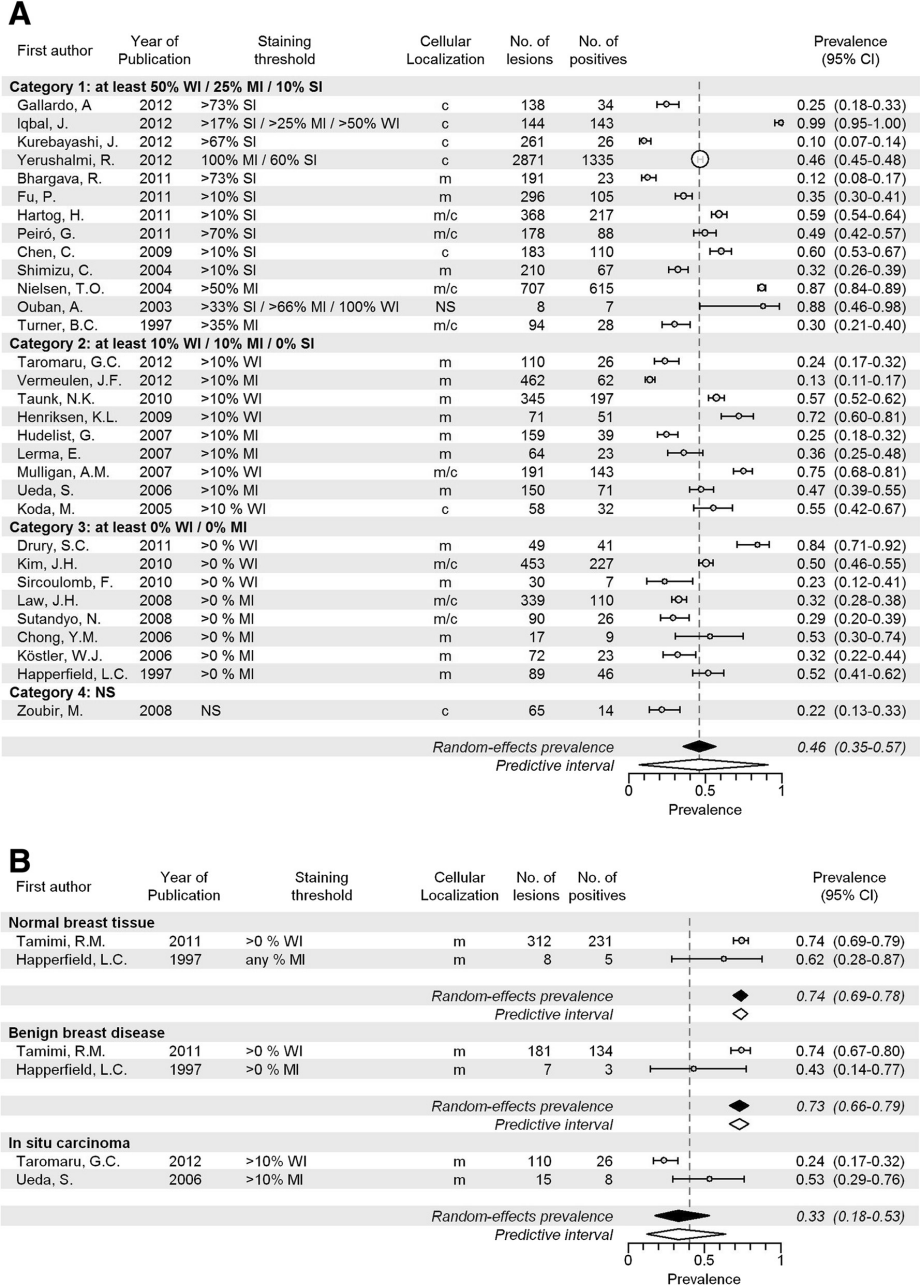
**Expression prevalence of IGF1R. A** Systematic literature review of IGF1R prevalence in breast cancer assessed by immunohistochemistry, according to reported staining threshold. Legend: Dashed gray reference line: overall random-effects prevalence estimate. Abbreviations: Staining threshold: weak intensity (WI), moderate intensity (MI), strong intensity (SI); Localization: cytoplasm (c), membrane (m); confidence interval (CI); not stated (NS). **B** Systematic literature review of IGF1R prevalence in normal breast tissue, benign breast diseases and carcinoma in situ assessed by immunohistochemistry. Legend: Dashed line represents random effect summary prevalence estimate for invasive cancer within studies reporting also on normal, benign and/or precancerous breast tissue ( 4 studies). Abbreviations: Staining threshold: weak intensity (WI), moderate intensity (MI), strong intensity (SI); Localization: cytoplasm (c), membrane (m); confidence interval (CI); not stated (NS).

We analyzed a total of 31 articles including 8,463 invasive cancers (range of 8 to 2,871 cancers per study). The pooled prevalence of IGF1R expression was 46% (CI 35-57%; Figure 5A and Table 2) with a range between studies of 10% to 99%. For studies investigating membranous staining patterns only, the pooled prevalence was 38% (CI 27-50%; 15 studies, Additional file 23: Figure S4A) and when the studies providing best evidence for evaluation of molecular imaging targets were selected, this was 33% (CI 22-46%; 10 studies, Additional file 24: Figure S4B). In contrast to the other investigated markers, the pooled prevalence of IGF1R was lower in grade III versus grade I cancers (41% versus 57%, p < 0.001; Additional file 25: Figure S4C), and was lower in T3 cancers compared to T1 cancers (39% versus 45%, p = 0.047; Additional file 26: Figure S4D). Prevalence of IGF1R was higher in IDC compared to ILC (42% versus 25%, p < 0.001; Additional file 27: Figure S4E), and higher in studies using TMAs than in studies using full sections (57% versus 34%, p = 0.032; Additional file 28: Figure S4F). In normal breast tissue, the pooled expression prevalence was 74% (CI 69-78%, p = 0.109; 2 studies). Pooled prevalence in benign lesions was 73% (CI 66-79%, p = 0.137; 2 studies), and in carcinoma *in situ* 33% (CI 18-53%, p = 0.869; 2 studies) (Figure 5B). Variation in results between studies was large, both overall and within the studies investigating membranous staining only and best evidence studies. Within groups of tumor grade and size, the between-study heterogeneity was very low, but the number of studies in these subgroups was small.

### Evaluation of publication bias

The substantial overall between-study heterogeneity in prevalence estimates was confirmed by examination of the funnel plots (not shown). Furthermore, smaller studies (i.e. with lower precision) were more likely to report higher hypoxia marker prevalence rates (all Egger’s tests p < 0.05, except for IGF1R). Funnel plots evaluating hypoxia marker prevalence rates according to tumor grade showed no evidence for publication bias for GLUT1 and CXCR4 (all Egger’s tests p > 0.25), but indicated that smaller studies showed a larger increase in CAIX prevalence for grade III versus I and a larger decrease in IGF1R prevalence for grade II versus grade I tumors (i.e. more extreme effects in small studies; Egger’s tests p = 0.044 and p = 0.023, respectively). We found no indication for publication bias when evaluating the studies reporting on hypoxia marker prevalence rates according to tumor size (all Egger’s tests p > 0.15, or too few studies for evaluation).

## Discussion

In this comprehensive systematic literature review and meta-analysis, we reported on expression prevalence of the hypoxia-related proteins GLUT1, CAIX, CXCR4, and IGF1R in breast cancer and carcinoma *in situ*, benign breast disease and normal breast tissue. We included a total of 117 articles totaling 30,216 IHC results. Hypoxia marker prevalence rates were in the range of other potential targets for molecular imaging with antibodies or antibody fragments, e.g. Epidermal Growth Factor Receptor (EGFR) [157] or CD44v6 [158], and were higher than for example Human Epidermal Growth Factor Receptor 2 (HER2) [159]. Benign breast disease and non-affected breast tissue showed low expression, but the number of available studies was limited. The between-study variation of results was substantial and meta-regression showed several clinicopathological features of breast cancer to significantly influence hypoxia marker expression.

CAIX, GLUT1, and CXCR4 prevalence rates significantly increased with histological grade, which is consistent with the hypothesis that high grade tumors have a higher proliferation rate, causing neo-angiogenesis to lag behind tumor growth [160]. The resulting inadequate nutrient and oxygen supply causes activation of the hypoxia pathway [161,162]. Surprisingly, we found an inverse relation for IGF1R with histological grade. In analogy to histological grade, larger tumors may also express hypoxia markers more frequently, but we only found a significant positive relation for CAIX, and again an unexpected negative significant relation with IGF1R. We further found that hypoxia proteins are infrequently expressed in ILC, suggesting that hypoxia is not a common phenomenon in these cancers. Ercan et al. reported that only 3% of ILC expresses HIF1α, compared to 39% of IDC [163], suggesting that hypoxia is indeed rare in this subtype. We found significant lower expression prevalences in normal tissue and benign breast diseases compared to invasive breast cancer, showing high cancer-specificity of CAIX, GLUT1 and especially CXCR4. IGF1R was evaluated in few studies with non-significant results, so no conclusions can be drawn with respect to specificity of this target. Pooled expression prevalence rates of carcinoma *in situ* were at least comparable to (GLUT1 and IGF1R) or higher than (CAIX and CXCR4) invasive carcinoma, albeit based on few studies.

An increasing number of recent studies evaluated hypoxia marker expression using TMA. Although TMA allows for higher throughput than full section analyses, it may lead to underestimation of marker expression in presence of intra-tumoral heterogeneity. We found that CAIX and GLUT1 prevalence was significantly lower in TMA studies, presumably due to the sampling method used for TMA construction, in which necrotic regions that usually have the highest expression of hypoxia-related proteins are avoided [65]. The lower prevalence of CAIX and GLUT1 in TMA studies could be interpreted as an indication of intratumoral heterogeneity of these markers. Although this interpretation is interesting, the applicability of TMAs for assessment of hypoxia marker expression needs to be reconsidered [164,165], even if their use has been justified for other markers (e.g. ERα and HER2 [143,166]).

With a view to molecular imaging, specificity of the imaging target is pivotal for every clinical application. The marked lower expression prevalence of CAIX, GLUT1 and CXCR4 in benign breast disease and normal breast tissue is thus highly promising. The specificity results for IGF1R are less encouraging, albeit based on only two studies. For early detection, a suitable target should be prevalent already in small tumors. Furthermore, evidence is mounting that current mammography screening may lead to substantial over-diagnosis [167] and picks-up tumors with favorable prognosis [168]. An imaging target that identifies small tumors with poor prognosis, e.g. grade III invasive breast cancers [169], would thus be especially valuable for screening. This combination makes GLUT1 an interesting candidate, with high expression in grade III cancers (58%) and also highest expression in T1 tumors (although the latter not significantly different from T3 tumors). CXCR4 and CAIX also show higher expression in grade III cancers, but for CAIX expression prevalence is markedly lower in smaller lesions. For intra-operative guidance, a high prevalence in invasive cancer is not required, as tissue can be sampled for investigation of target expression pre-operatively. Such a target should ideally show low intratumoral heterogeneity to ascertain radical resection. Although this was not specifically addressed by individual studies, the difference in results between TMA and full-section studies may indicate that CAIX and GLUT1 have marked intratumoral heterogeneity and might therefore be less suitable for intra-operative application than CXCR4. Risk factors for incomplete tumor resection in current clinical practice include the presence of an extensive intraductal component [170], and the ILC histological subtype [171-175]. Especially CXCR4 shows high expression in DCIS, thus is potentially valuable for imaging of an extensive intraductal component. However, none of the markers show potential for ILC imaging.

None of the investigated markers showed a sufficient expression prevalence to allow sensitive molecular imaging with a single tracer only. For successful implementation (especially in a screening or diagnostic setting), a combination of tracers would be required to obtain a high sensitivity. However, all of the investigated markers here are expressed via the same (hypoxia-related) oncogenic pathway. The few studies that investigated co-expression patterns found that expression of these markers were indeed closely correlated. It would therefore be more advantageous to combine the hypoxia targets with targets from other oncogenic pathways, such as growth factors (e.g. EGFR or HER2), targets that are excreted in the tumor stroma (e.g. Vascular Endothelial Growth Factor (VEGF)), or less tumor-specific targets such as Mucin 1 (MUC1), Mammaglobin, or CD44v6 [36]. However, the aggregated nature of the obtained data did not allow us to investigate the best combination of targets or to investigate co-expression patterns.

To appreciate the results, one needs to acknowledge that studies employed various IHC protocols and assessment methodologies, as no standardized scoring system is established for these markers, in contrast to e.g. HER2 [176]. When we evaluated only studies that used relatively strict cut-offs (i.e. the studies providing best evidence for evaluation of molecular imaging targets) or studies investigating membranous staining only, the results were still comparable to the overall results. Nevertheless, as IHC may not reflect the functionality or availability of a marker in all situations, it remains unclear which or if any cut-off level relates to sufficient marker levels for molecular imaging in humans [177]. However, IHC remains the established standard for protein expression estimation since it allows for sensitive detection at the (sub-) cellular level, and is more reliable than assays measuring DNA or RNA levels because of post-translational processing.

## Conclusions

We have shown that human expression prevalence and patterns of hypoxia-related markers support their potential as molecular imaging targets, with promising specificity. However, none of the evaluated markers shows sufficient prevalence in invasive cancer to be exploited as the sole target. Future research should focus on the identification of optimal combinations of candidate imaging targets, and dedicated studies are needed to assess the accuracy of such combinations to discriminate between breast cancer (subtypes) and benign breast lesions and normal tissue. The data from this review and such studies could help guide the choice of markers for breast cancer tracer development.

## Competing interests

The authors declare that they have no competing interests.

## Authors’ contributions

AA, AB, JV, WM, EvW, PvD, and SE conceived and designed the study. AA, AvB, and JV selected the articles. AA, AvB, JV, and SE analyzed the data. AA, AvB and JV wrote the first draft of the manuscript. AA, AvB, JV, WM, EvW, PvD, and SE contributed to the writing of the manuscript, agree with the results and conclusions. All authors read and approved the final manuscript.

## Pre-publication history

The pre-publication history for this paper can be accessed here:

http://www.biomedcentral.com/1471-2407/13/538/prepub

## Supplementary Material

Additional file 1: Table S1A Study characteristics of articles included in the review investigating CAIX expression prevalences in breast cancer, carcinoma *in situ,* benign breast disease, and normal breast tissue Legend: a: mean; *: mean size in mm (range or SD); b: nuclear grade; ns: not stated; na: not applicable; ○ : tissue not investigated; ●: tissue investigated; ◉: both TMA and full sections investigated; IDC: invasive ductal carcinoma; ILC: invasive lobular carcinoma; IBC: inflammatory breast cancer; ABC: advanced breast cancer; TN: triple negative; LN: lymph node; TMA: tissue microarray; PT: phyllodes tumors; FA: fibroadenoma; M: distant metastasis; Obs.: number of observers evaluating staining result; +: positive; -: negative. Click here for file

Additional file 2: Table S1B Study characteristics of articles included in the review investigating GLUT1 expression prevalences in breast cancer, carcinoma *in situ*, benign breast disease, and normal breast tissue. Legend: a: mean; *: mean size in mm (range or SD); b: nuclear grade; ns: not stated; na: not applicable; ○ : tissue not investigated; ●: tissue investigated; ◉: both TMA and full sections investigated; IDC: invasive ductal carcinoma; ILC: invasive lobular carcinoma; IBC: inflammatory breast cancer; ABC: advanced breast cancer; TN: triple negative; LN: lymph node; TMA: tissue microarray; PT: phyllodes tumors; FA: fibroadenoma; M: distant metastasis; Obs.: number of observers evaluating staining result; +: positive; -: negative. Click here for file

Additional file 3: Table S1C Study characteristics of articles included in the review investigating CXCR4 expression prevalences in breast cancer, *in situ* carcinoma, benign breast disease, and normal breast tissue. Legend: a: mean; *: mean size in mm (range or SD); b: nuclear grade; ns: not stated; na: not applicable; ○ : tissue not investigated; ●: tissue investigated; ◉: both TMA and full sections investigated; IDC: invasive ductal carcinoma; ILC: invasive lobular carcinoma; IBC: inflammatory breast cancer; ABC: advanced breast cancer; TN: triple negative; LN: lymph node; TMA: tissue microarray; PT: phyllodes tumors; FA: fibroadenoma; M: distant metastasis; Obs.: number of observers evaluating staining result; +: positive; -: negative. Click here for file

Additional file 4: Table S1D Study characteristics of articles included in the review investigating IGF1R expression prevalences in breast cancer, carcinoma *in situ*, benign breast disease, and normal breast tissue. Legend: a: mean; *: mean size in mm (range or SD); b: nuclear grade; ns: not stated; na: not applicable; ○ : tissue not investigated; ●: tissue investigated; ◉: both TMA and full sections investigated; IDC: invasive ductal carcinoma; ILC: invasive lobular carcinoma; IBC: inflammatory breast cancer; ABC: advanced breast cancer; TN: triple negative; LN: lymph node; TMA: tissue microarray; PT: phyllodes tumors; FA: fibroadenoma; M: distant metastasis; Obs.: number of observers evaluating staining result; +: positive; -: negative. Click here for file

Additional file 5: Figure S1A CAIX - Membranous staining. Systematic literature review of CAIX prevalence in breast cancer assessed by immunohistochemistry for studies investigating membranous staining patterns only. Click here for file

Additional file 6: Figure S1B CAIX - Best evidence studies. Systematic literature review of CAIX prevalence in breast cancer assessed by immunohistochemistry for studies providing the best evidence for high expression levels. Click here for file

Additional file 7: Figure S1C CAIX - Histological grade. Systematic literature review of CAIX prevalence in breast cancer assessed by immunohistochemistry in relation to histological grade. Click here for file

Additional file 8: Figure S1D CAIX - Tumor size. Systematic literature review of CAIX prevalence in breast cancer assessed by immunohistochemistry in relation to tumor size. Click here for file

Additional file 9: Figure S1E CAIX - Histology. Systematic literature review of CAIX prevalence in breast cancer assessed by immunohistochemistry in relation to histological subtype. Click here for file

Additional file 10: Figure S1F CAIX - Specimen handling. Systematic literature review of CAIX prevalence in breast cancer assessed by immunohistochemistry in relation to specimen handling method. Click here for file

Additional file 11: Figure S2A GLUT1 - Membranous staining. Systematic literature review of GLUT1 prevalence in breast cancer assessed by immunohistochemistry for studies investigating membranous staining patterns only. Click here for file

Additional file 12: Figure S2B GLUT1 - Best evidence studies. Systematic literature review of GLUT1 prevalence in breast cancer assessed by immunohistochemistry for studies providing the best evidence for high expression levels. Click here for file

Additional file 13: Figure S2C GLUT1 - Histological grade. Systematic literature review of GLUT1 prevalence in breast cancer assessed by immunohistochemistry in relation to histological grade. Click here for file

Additional file 14: Figure S2D GLUT1 - Tumor size. Systematic literature review of GLUT1 prevalence in breast cancer assessed by immunohistochemistry in relation to tumor size. Click here for file

Additional file 15: Figure S2E GLUT1 - Histology. Systematic literature review of GLUT1 prevalence in breast cancer assessed by immunohistochemistry in relation to histological subtype. Click here for file

Additional file 16: Figure S2F GLUT1 - Specimen handling. Systematic literature review of GLUT1 prevalence in breast cancer assessed by immunohistochemistry in relation to specimen handling method. Click here for file

Additional file 17: Figure S3A CXCR4 - Membranous staining. Systematic literature review of CXCR4 prevalence in breast cancer assessed by immunohistochemistry for studies investigating membranous staining patterns only. Click here for file

Additional file 18: Figure S3B CXCR4 - Best evidence studies. Systematic literature review of CXCR4 prevalence in breast cancer assessed by immunohistochemistry for studies providing the best evidence for high expression levels. Click here for file

Additional file 19: Figure S3C CXCR4 - Histological grade. Systematic literature review of CXCR4 prevalence in breast cancer assessed by immunohistochemistry in relation to histological grade. Click here for file

Additional file 20: Figure S3D CXCR4 - Tumor size. Systematic literature review of CXCR4 prevalence in breast cancer assessed by immunohistochemistry in relation to tumor size. Click here for file

Additional file 21: Figure S3E CXCR4 - Histology. Systematic literature review of CXCR4 prevalence in breast cancer assessed by immunohistochemistry in relation to histological subtype. Click here for file

Additional file 22: Figure S3F CXCR4 - Specimen handling. Systematic literature review of CXCR4 prevalence in breast cancer assessed by immunohistochemistry in relation to specimen handling method. Click here for file

Additional file 23: Figure S4A IGF1R - Membranous staining. Systematic literature review of IGF1R prevalence in breast cancer assessed by immunohistochemistry for studies investigating membranous staining patterns only. Click here for file

Additional file 24: Figure S4B IGF1R - Best evidence studies. Systematic literature review of IGF1R prevalence in breast cancer assessed by immunohistochemistry for studies providing the best evidence for high expression levels. Click here for file

Additional file 25: Figure S4C IGF1R - Histological grade. Systematic literature review of IGF1R prevalence in breast cancer assessed by immunohistochemistry in relation to histological grade. Click here for file

Additional file 26: Figure S4D IGF1R - Tumor size. Systematic literature review of IGF1R prevalence in breast cancer assessed by immunohistochemistry in relation to tumor size. Click here for file

Additional file 27: Figure S4E IGF1R - Histology. Systematic literature review of IGF1R prevalence in breast cancer assessed by immunohistochemistry in relation to histological subtype. Click here for file

Additional file 28: Figure S4F IGF1R - Specimen handling. Systematic literature review of IGF1R prevalence in breast cancer assessed by immunohistochemistry in relation to specimen handling method. Click here for file
