# Supplementary material for: The potential of hypoxia markers as target for breast molecular imaging – a systematic review and meta-analysis of human marker expression
Source: BMC Cancer. 2013 Nov 10;13:538. doi: 10.1186/1471-2407-13-538 (PMC3903452; doi:10.1186/1471-2407-13-538)
Supplement: Additional file 2: Table S1B — Study characteristics of articles included in the review investigating GLUT1 expression prevalences in breast cancer, carcinoma in situ, benign breast disease, and normal breast tissue. Legend: a: mean; *: mean size in mm (range or SD); b: nuclear grade; ns: not stated; na: not applicable; ○ : tissue not investigated; ●: tissue investigated; ◉: both TMA and full sections investigated; IDC: invasive ductal carcinoma; ILC: invasive lobular carcinoma; IBC: inflammatory breast cancer; ABC: advanced breast cancer; TN: triple negative; LN: lymph node; TMA: tissue microarray; PT: phyllodes tumors; FA: fibroadenoma; M: distant metastasis; Obs.: number of observers evaluating staining result; +: positive; -: negative. [file 1471-2407-13-538-S2.pdf]

Additional Information Table S1C. Study characteristics of articles included in the review investigating CXCR4 expression prevalences in breast cancer, *in situ* carcinoma, benign breast disease, and normal breast tissue.

**Reference**			**Study population**		**Patient and tumor characteristics**			**Histology**							**IHC**				
First author<br>Year, Country of patient inclusion	Inclusion period	No. of invasive cancers (+other tissue)	Important selection criteria	Age (range/ SD)	Tumor size* pT (0% – 100%)	Histological grade (0% – 100%)	LN[+] (%)	Normal	Benign	In situ	invasive	IDC	ILC	Other	TMA	Cores	(mm)	Antibody (manufacturer)	Obs.
**Huang, S.[92]**<br>2012, China	2002-2007	197 (+20)	-	44(23-75)	1<br>2/3	I<br>II<br>III	56	○	●	○	○	●	○	○	○			12G5 (Abcam)	ns
**Jin, F.[93]**<br>2012, Germany	1989-1993	140	-	ns	ns	ns	ns	○	○	○	●	○	○	○	○			na (na)	ns
**Ibrahim, T.[94]**<br>2011, Italy,	1997-2000	40	-	66[a](42-87)	ns	I<br>II<br>III	ns	○	○	○	○	●	○	○	○			Ab58176 (Abcam)	2
**Sacanna, E.[95]**<br>2011, Italy	1997-2000	40	-	66[a](42-87)	ns	I<br>II<br>III	ns	○	○	○	○	●	○	○	○			Ab58176 (Abcam)	2
**Bohn, O.L.[96]**<br>2010, USA	ns	64	M-	ns	1<br>2<br>3	ns	41	○	○	○	○	●	●	●	●	3	0.6	Ab2074 (Abcam)	1
**Kobayashi, T.[97]**<br>2010, Japan	ns	223	-	52(30-82)	1/2<br>3	I[b]<br>II<br>II	45	○	○	○	○	●	●	●	●	2	2.0	MAB173 (R&D Systems)	2
**Liu, Y.[98]**<br>2010, China	1997-2004	200	-	51(37-74)	1<br>2<br>3	I<br>II<br>III	50	○	○	○	○	●	○	○	●	2	2.0	ns (Abcam)	2
**Andre, F.[99]**<br>2009, France	1989-1995	794	grade 2-3 or LN+	56[a](ns)	<3 cm<br>≥3 cm	I<br>II<br>III	43	○	○	○	●	○	○	○	●	3	ns	MAB172 (R&D systems)	2
**Cabioglu, N.[100]**<br>2009, USA	ns	29	M+	44(27-79)	ns	I<br>II<br>III	ns	○	○	○	○	●	●	●	○			MAB173 (R&D systems)	ns
**Hassan, S.[101]**<br>2009, Canada	2000-2003	236	stage I-III	ns	1<br>2<br>3	I<br>II<br>III	46	○	○	○	●	○	○	○	●	2/3	ns	TN14003 (na)	ns
**Kim, J.O.[102]**<br>2009, South Korea	2000-2009	44	-	52[a](28-77)	1<br>2<br>3	I<br>II<br>III	41	○	○	○	○	●	○	○	●	ns	5.0	ns (Abcam)	ns
**Liu, F.[103]**<br>2009, China	2004-2007	199	Including micro-papillary carc.	53[a](29-83)	1<br>2<br>3	I<br>II<br>III	70	○	○	○	○	●	○	●	○			MAB172 (R&D systems)	ns
**Yasuoka, H.[104]**	1981-1992	113	family history -	51(24-87)	1	I/II	52	○	○	○	○	●	○	●	○			ns (Abcam)	3

2009, Japan					2/3		III														
**Zhou, W.[105]** 2009, China	2004-2006	63 (+20)	Stage ≤IIIA	53[a](32-80)	1 2 3		I/II III		46	●	○	○	○	●	○	●	○			MAB172 (R&D systems)	2
**Blot, E.[106]** 2008, France	1991-1991	194	-	57[a](26-84)	1 2/3		I II/III		56	○	○	○	●	○	○	○	○			12G5 (R&D systems)	ns
**Fujii, T.[107]** 2008, Japan	1993-1999	73	-	56[a](11)	1 2/3		I II III		46	○	○	○	●	○	○	○	○			ns (Prosci Inc.)	2
**Woo, S.[108]** 2008, South Korea	1997-2002	105	M-	50(28-78)	1 2 3		I II III		52	○	○	○	●	○	○	○	○			MAB172 (R&D systems)	2
**Yasuoka, H.[109]** 2008, Japan	1981-1992	113	family history -	51(24-87)	1 2/3		I/II III		52	○	○	○	○	●	○	●	○			ns (Abcam)	3
**Hao, L.[110]** 2007, China	2002-2005	76 (+14)	-	50(ns)	1 2/3		I II III		54	●	●	○	●	○	○	○	○			MAB172 (R&D systems)	2*
**Tsoli, E.[111]** 2007, UK	1999-2001	151	-	63(29-94)	ns		I II III		38	○	○	○	○	●	●	○	●	4	3.0	MAB171 (R&D systems)	ns
**Andre, F.[112]** 2006, France	1972-1979	133	LN+	50(29-64)		25 (10-90)	I/II III		100	○	○	○	●	○	○	○	○			MAB172 (R&D systems)	2
**Kim, R.[113]** 2006, Japan	1980-2001	22	M+ (bone)	55(23-79)	ns		ns		69	○	○	○	○	●	○	○	○			ns (Abcam)	ns
**Salvucci, O.[114]** 2006, Switzerland	1985-2001	1808 (+214)	-	62(26-101)	ns		I II III		48	●	●	●	○	●	●	●	●	ns	0.6	MAB172 (R&D systems)	2
**Shim, H.[115]** 2006, USA	ns	94 (+44)	-	ns	1/2 3		ns		21	●	○	○	●	○	○	○	●	ns	ns	TN14003 (na)	ns
**Su, Y.C.[116]** 2006, Taiwan	1998-2004	85	pT1-2, clinically LN-	44[a](ns)	1 2 3		I II III		31	○	○	○	●	○	○	○	○			MAB172 (R&D systems)	2
**Cabioglu, N.[117]** 2005, Turkey	2000-2001	18	clinically LN-	45(38-76)	1 2/3		ns		67	○	○	○	○	●	○	●	○			MAB173 (R&D systems)	ns
**Cabioglu, N. 2[118]** 2005, USA	ns	197	pT1	ns	1 2 3		ns		50	○	○	○	●	○	○	○	○			MAB173 (R&D systems)	ns
**Li, Y.M.[119]** 2004, USA	ns	219	-	ns	ns		ns		ns	○	○	○	●	○	○	○	○			MAB170 (R&D systems)	2
**Schmid, B.C.[120]** 2004, Austria	2000-2001	7 (+28)	pT1-2	ns	1/2 3		ns		ns	○	●	●	○	●	○	○	○			12G5 (R&D systems)	ns
**Kato, M.[121]** 2003, Japan	1990-1996	59	-	53[a](9)	1 2 3		I II III		49	○	○	○	○	●	○	○	○			ns (BD Pharmingen)	2

a: mean; *: mean size in mm (range or SD); b: nuclear grade; ns: not stated; na: not applicable; ○: tissue not investigated; ●: tissue investigated; ⦿:both TMA and full sections investigated; IDC: invasive ductal carcinoma; ILC: invasive lobular carcinoma; IBC: inflammatory breast cancer; ABC: advanced breast cancer; TN: triple negative; LN: lymph node; TMA: tissue microarray PT: phyllodes tumors FA: fibroadenoma; M: distant metastasis, Obs.: number of observers evaluating staining result, +: positive; -: negative
