# Supplementary material for: The potential of hypoxia markers as target for breast molecular imaging – a systematic review and meta-analysis of human marker expression
Source: BMC Cancer. 2013 Nov 10;13:538. doi: 10.1186/1471-2407-13-538 (PMC3903452; doi:10.1186/1471-2407-13-538)
Supplement: Additional file 3: Table S1C — Study characteristics of articles included in the review investigating CXCR4 expression prevalences in breast cancer, in situ carcinoma, benign breast disease, and normal breast tissue. Legend: a: mean; *: mean size in mm (range or SD); b: nuclear grade; ns: not stated; na: not applicable; ○ : tissue not investigated; ●: tissue investigated; ◉: both TMA and full sections investigated; IDC: invasive ductal carcinoma; ILC: invasive lobular carcinoma; IBC: inflammatory breast cancer; ABC: advanced breast cancer; TN: triple negative; LN: lymph node; TMA: tissue microarray; PT: phyllodes tumors; FA: fibroadenoma; M: distant metastasis; Obs.: number of observers evaluating staining result; +: positive; -: negative. [file 1471-2407-13-538-S3.pdf]

Additional Information Table S1D. Study characteristics of articles included in the review investigating IGF1R expression prevalences in breast cancer, *in situ* carcinoma, benign breast disease, and normal breast tissue.

Reference			Study population		Patient and tumor characteristics					Histology							IHC				
First author<br>Year, Country of patient inclusion	Inclusion period	No. of invasive cancers (+other tissue)	Important selection criteria	Age (range/ SD)	Tumor size* pT	0% 100%	Histological grade	0% 100%	LN$^+$ (%)	Normal	Benign	In situ	invasive	IDC	ILC	Other	TMA	Cores	(mm)	Antibody (manufacturer)	Obs.
**Gallardo, A.[122]**<br>2012, Spain	ns	138	HER2+	55 (31-92)		25 (10-200)	I<br>II<br>III		57	○	○	○	●	○	○	○	●	3	ns	24-31 (Neomarkers)	3
**Fu, P.[123]**<br>2012, Japan	2001-2009	77	-	ns	1<br>2/3		I/II [b]<br>III		35	○	○	○	●	○	○	○	○			3027 (Cell Signaling)	ns
**Iqbal J.[124]**<br>2012, Singapore	ns	144	TN	53 (28-88)	1<br>2/3		I<br>II<br>III		28	○	○	○	○	●	●	●	●	2	1.0	Clone 3027 (Cell Signaling)	2
**Kurebayashi, J.[125]**<br>2012, Japan	1999-2003	261	-	59 (ns)	1<br>2/3		I<br>II<br>III		20	○	○	○	●	○	○	○	○			24-31 (Millipore)	1
**Taromaru, G.C.[126]**<br>2012, Brazil	2002-2008	110 (+110)	including DCIS	55 (26-90)	ns		ns		ns	○	○	●	○	●	○	○	○			24-31 (Pleasanton)	2
**Yerushalmi, R.[127]**<br>2012, Canada	1986-1992	2871	-	ns	1<br>2<br>3		I<br>II<br>III		46	○	○	○	●	○	○	○	●	ns	0.6	Sc-713 (Santa Cruz)	1
**Vermeulen, J.F.[36]**<br>2012, The Netherlands	1997-2007	462	-	60[a] (28-88)	1<br>2<br>3		I<br>II<br>III		48	○	○	○	○	●	●	●	●	3	0.6	NB110-87052 (Novus)	1
**Bhargava, R.[128]**<br>2011, USA	ns	191	-	ns	ns		ns		ns	○	○	○	○	●	●	●	●	2	ns	G11 (Ventana)	ns
**Drury, S.C.[129]**<br>2011, UK	1981-2004	49	recurrence	55 (28-81)		20 (3-70)	I<br>II<br>III		23	○	○	○	●	○	○	○	●	2*3	0.6	G11 (Ventana)	2
**Fu, P.[130]**<br>2011, Japan	2001-2009	296	-	59 (21-93)	1<br>2/3		I/II [b]<br>III		39	○	○	○	●	○	○	○	○			Clone 3027 (Cell Signaling)	ns
**Hartog, H.[131]**<br>2011, The Netherlands	1996-2005	368	-	59 (27-91)	1<br>2<br>3		I<br>II<br>III		46	○	○	○	○	●	○	○	●	ns	ns	24-31 (Calbiochem)	1
**Peiró, G.[132]**<br>2011, Spain	1990-2001	178	LN-, stage I-II	52[a] (23-88)	1<br>2<br>3		I<br>II<br>III		0	○	○	○	○	●	●	○	●	ns	0.6	ns (Neomarkers)	2
**Tamimi, R.M.[133]**<br>2011, USA	1976-1996	0 (+493)	-	46 [a] (9)	na		na		na	●	●	○	○	○	○	○	●	3	0.6	24-31 (Lab Vision)	ns
**Kim, J.H.[134]**	2000-2006	453	-	46 (23-84)	1<br>2		I<br>II		42	○	○	○	●	○	○	○	●	ns	6.0	ns (Cell	ns

2010, South Korea					3		III													Signaling)	
**Sircoulomb, F.[135]** 2010, France	1987-2007	30	HER2+	ns	1<br>2<br>3		I<br>II<br>III		69	○	○	○	●	○	○	○	○			G11 (Ventana)	2
**Taunk, N.K.[136]** 2010, USA	1975-2003	345	-	ns	1<br>2<br>3		ns		6	○	○	○	●	○	○	○	●	ns	ns	T8C8 (Abcam)	1
**Chen, C.[137]** 2009,USA	1983-1998	183	-	58[a](26-89)	ns		ns		ns	○	○	○	○	●	●	○	○			Sc-462 (Santa Cruz)	2
**Henriksen, K.L.[138]** 2009, Denmark	ns	71	postmenopausal, ABC	65(44-84)	ns		ns		ns	○	○	○	○	●	●	●	●	ns	2.0	24-31 (Neomarkers)	1
**Peiró, G.[139]** 2008, Spain	1990-1999	165	LN-, stage I-II	52[a](23-88)	1<br>2<br>3		I<br>II/III		0	○	○	○	○	●	●	○	●	ns	0.6	ns (Neomarkers)	2
**Law, J.H.[140]** 2008, Canada	1974-1995	339	-	61[a](28-87)	1<br>2/3		ns		32	○	○	○	●	○	○	○	●	2	0.6	C20 (Santa Cruz)	ns
**Sutandyo, N.[141]** 2008, Indonesia	2004-2005	90	-	ns(24-75)	1<br>2<br>3		I<br>II<br>III		60	○	○	○	○	●	●	●	○			RB-9240-R7 (Labvision)	ns
**Zoubir, M.[142]** 2008, France	1995-2005	65	-	79(57-98)		42 (15-90)	I<br>II<br>III		ns	○	○	○	○	●	●	●	●	2	ns	Clone 3027 (Cell Signaling)	ns
**Henriksen, K.L.[143]** 2007, Denmark	ns	54	-	ns	ns		ns		ns	○	○	○	○	●	●	○	●	ns	2.0	24-31 (Neomarkers)	1
**Hudelist, G.[144]** 2007, Austria	ns	159	including BRCA1/BRCA2	ns(27-85)	ns		I<br>II<br>III		ns	○	○	○	○	●	●	●	○			24-31 (Neomarkers)	2
**Lerma, E.[145]** 2007,Spain	2000-2006	64	TN	53[a](25-86)	1<br>2<br>3		I<br>II<br>III		50	○	○	○	○	●	○	●	○			ns (Cell signaling)	2
**Mulligan, A.M.[146]** 2007, Canada	1989-1996	191	-	52[a](28-74)	1<br>2<br>3		I<br>II<br>III		34	○	○	○	●	○	○	○	●	2	0.6	24-31 (Oncogene)	ns
**Chong, Y.M.[147]** 2006, UK	1997-1998	17	-	69(ns)	ns		I<br>II<br>III		ns	○	○	○	●	○	○	○	○			αIR3 (Sigma Aldrich)	1
**Köstler, W.J.[148]** 2006, Austria	1999-2002	72	HER2+	53(27-83)	ns		I<br>II<br>III		ns	○	○	○	○	●	●	●	○			24-31 (Neomarkers)	2
**Ueda, S.[149]** 2006, Japan	1995-1997	150 (+15)	-	54(30-81)	1<br>2/3		I[b]<br>II<br>III		35	○	○	●	○	●	●	○	●	ns	2.0	ns (Neomarkers)	ns
**Koda, M.[150]** 2005, Poland	ns	58	-	55[a](30-80)	ns		I<br>II<br>III		52	○	○	○	○	●	○	○	○			H-60 (Santa Cruz)	ns
**Nielsen, T.O.[151]** 2004, Canada	1976-1990	707	-	46(22-86)	1<br>2		ns		60	○	○	○	●	○	○	○	●	1	0.6	C-20 (Santa Cruz)	ns

					3															
**Shimizu, C.[152]** 2004, Japan	1997-1997	210	-	53(25-83)		22 (1-140)	I II III	45	○	○	○	○	●	●	●	○			24-31 (ns)	ns
**Koda, M.[153]** 2003, Poland	2000-2002	50	-	55[a](30-80)	ns		I II III	49	○	○	○	○	●	○	○	○			H-60 (Santa Cruz)	2
**Ouban, A.[154]** 2003, USA	ns	8	-	63(18-93)	ns		ns	ns	○	○	○	○	●	●	○	●	ns	ns	C-20 (Santa Cruz)	2
**Happerfield, L.C.[155]** 1997, UK	ns	89 (+15)	-	ns	ns		I II III	ns	●	●	○	○	●	●	○	○			αIR3 (na)	ns
**Turner, B.C.[156]** 1997, USA	ns	94	-	ns		19 (ns)	ns	11	○	○	○	○	●	○	○	○			ns (Santa Cruz)	1

a: mean; *: mean size in mm (range or SD); b: nuclear grade; ns: not stated; na: not applicable; ○: tissue not investigated; ●: tissue investigated; ⦿:both TMA and full sections investigated; IDC: invasive ductal carcinoma; ILC: invasive lobular carcinoma; IBC: inflammatory breast cancer; ABC: advanced breast cancer; TN: triple negative; LN: lymph node; TMA: tissue microarray PT: phyllodes tumors FA: fibroadenoma; M: distant metastasis, Obs.: number of observers evaluating staining result, +: positive; -: negative
