# Supplementary figures and images for: The potential of hypoxia markers as target for breast molecular imaging – a systematic review and meta-analysis of human marker expression
Source: BMC Cancer. 2013 Nov 10;13:538. doi: 10.1186/1471-2407-13-538 (PMC3903452; doi:10.1186/1471-2407-13-538)

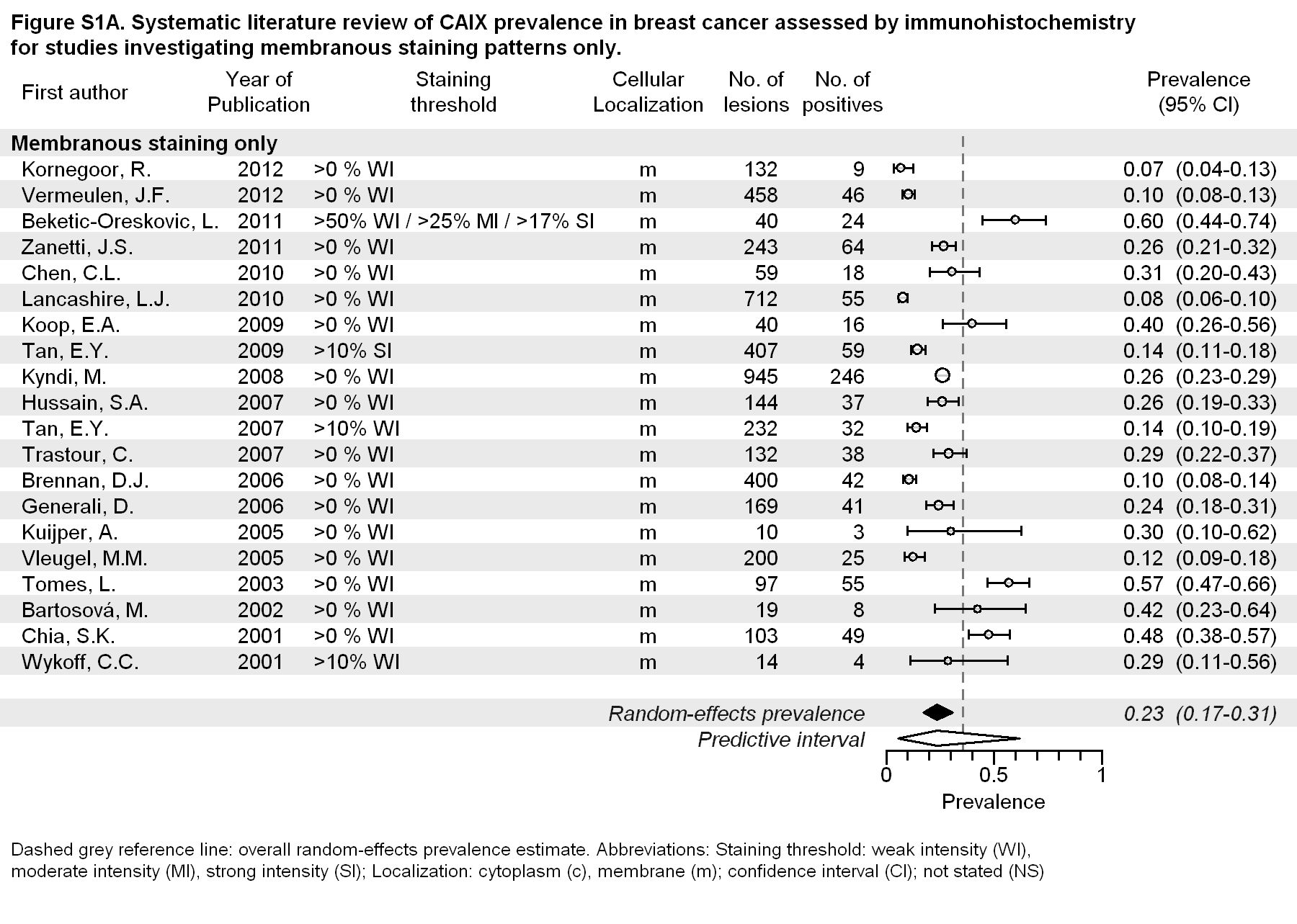

Supplement: Additional file 5: Figure S1A — CAIX - Membranous staining. Systematic literature review of CAIX prevalence in breast cancer assessed by immunohistochemistry for studies investigating membranous staining patterns only. [file 1471-2407-13-538-S5.jpeg]

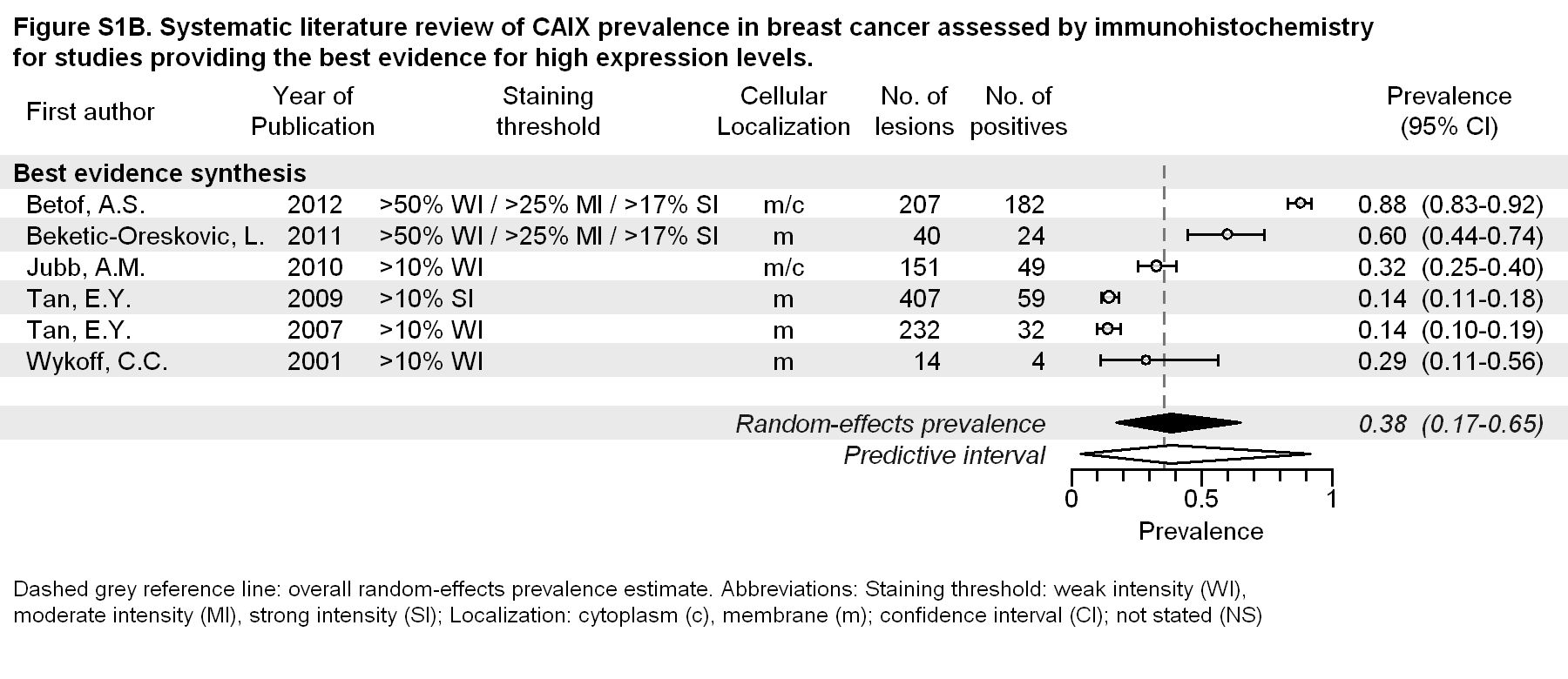

Supplement: Additional file 6: Figure S1B — CAIX - Best evidence studies. Systematic literature review of CAIX prevalence in breast cancer assessed by immunohistochemistry for studies providing the best evidence for high expression levels. [file 1471-2407-13-538-S6.jpeg]

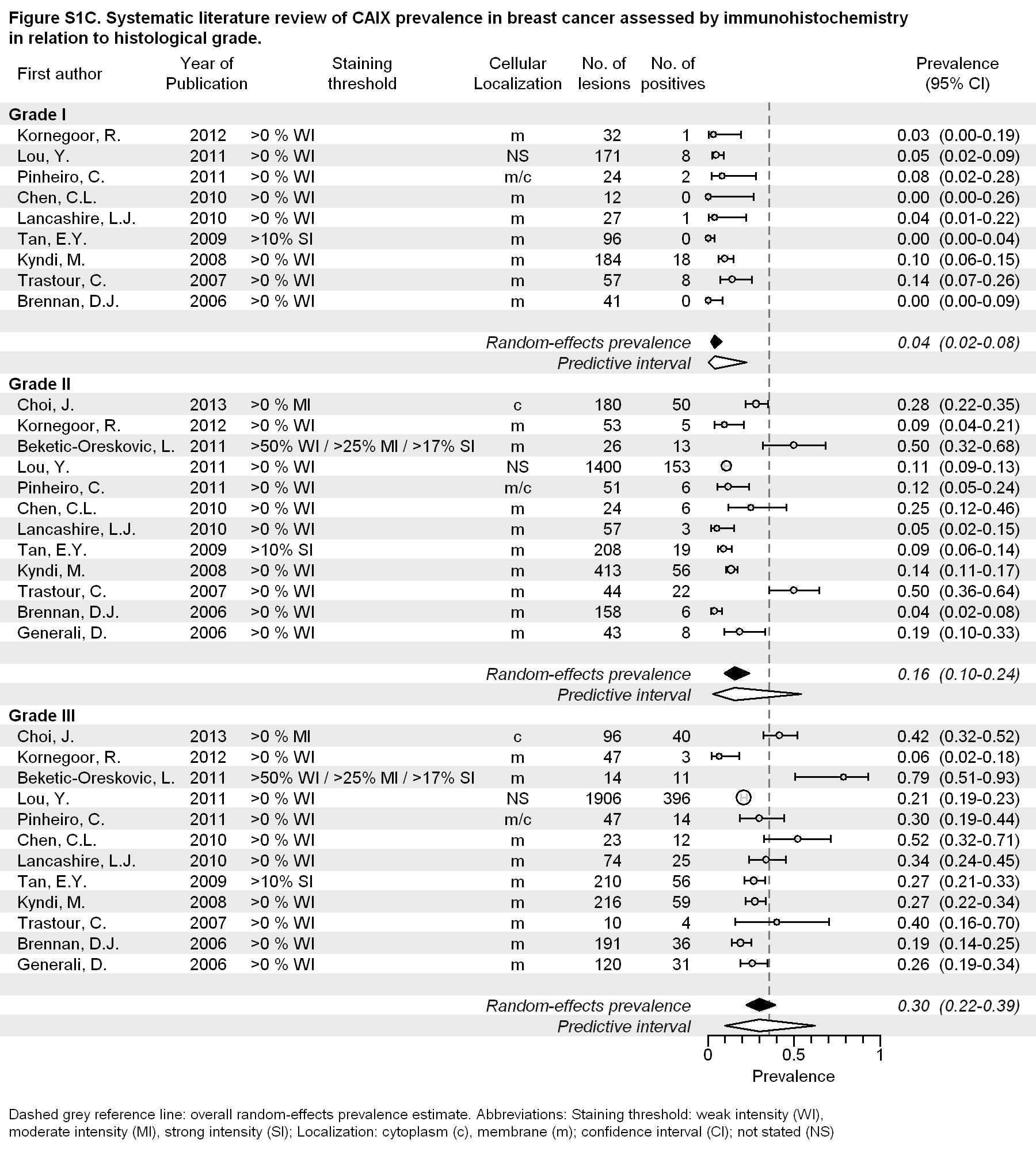

Supplement: Additional file 7: Figure S1C — CAIX - Histological grade. Systematic literature review of CAIX prevalence in breast cancer assessed by immunohistochemistry in relation to histological grade. [file 1471-2407-13-538-S7.jpeg]

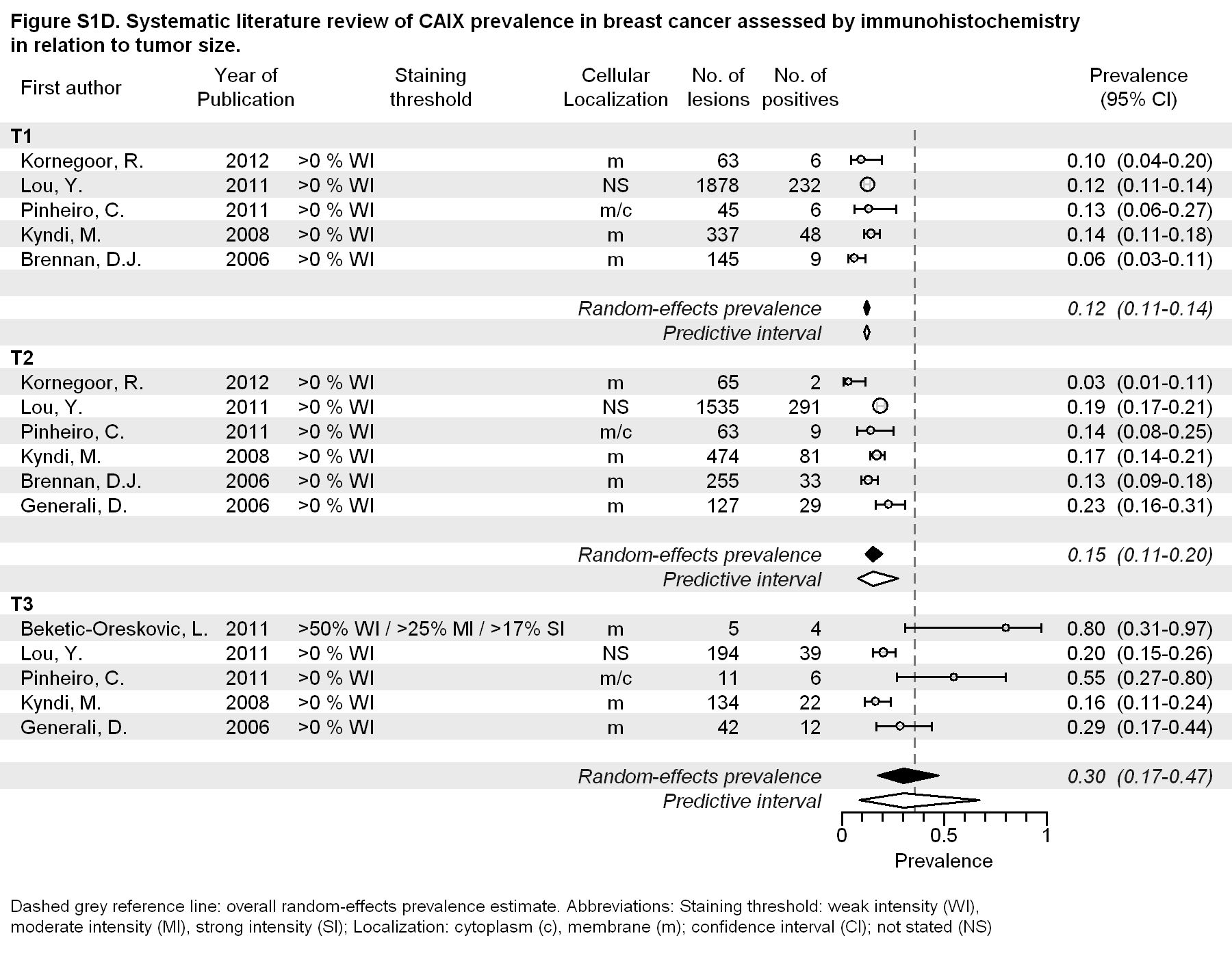

Supplement: Additional file 8: Figure S1D — CAIX - Tumor size. Systematic literature review of CAIX prevalence in breast cancer assessed by immunohistochemistry in relation to tumor size. [file 1471-2407-13-538-S8.jpeg]

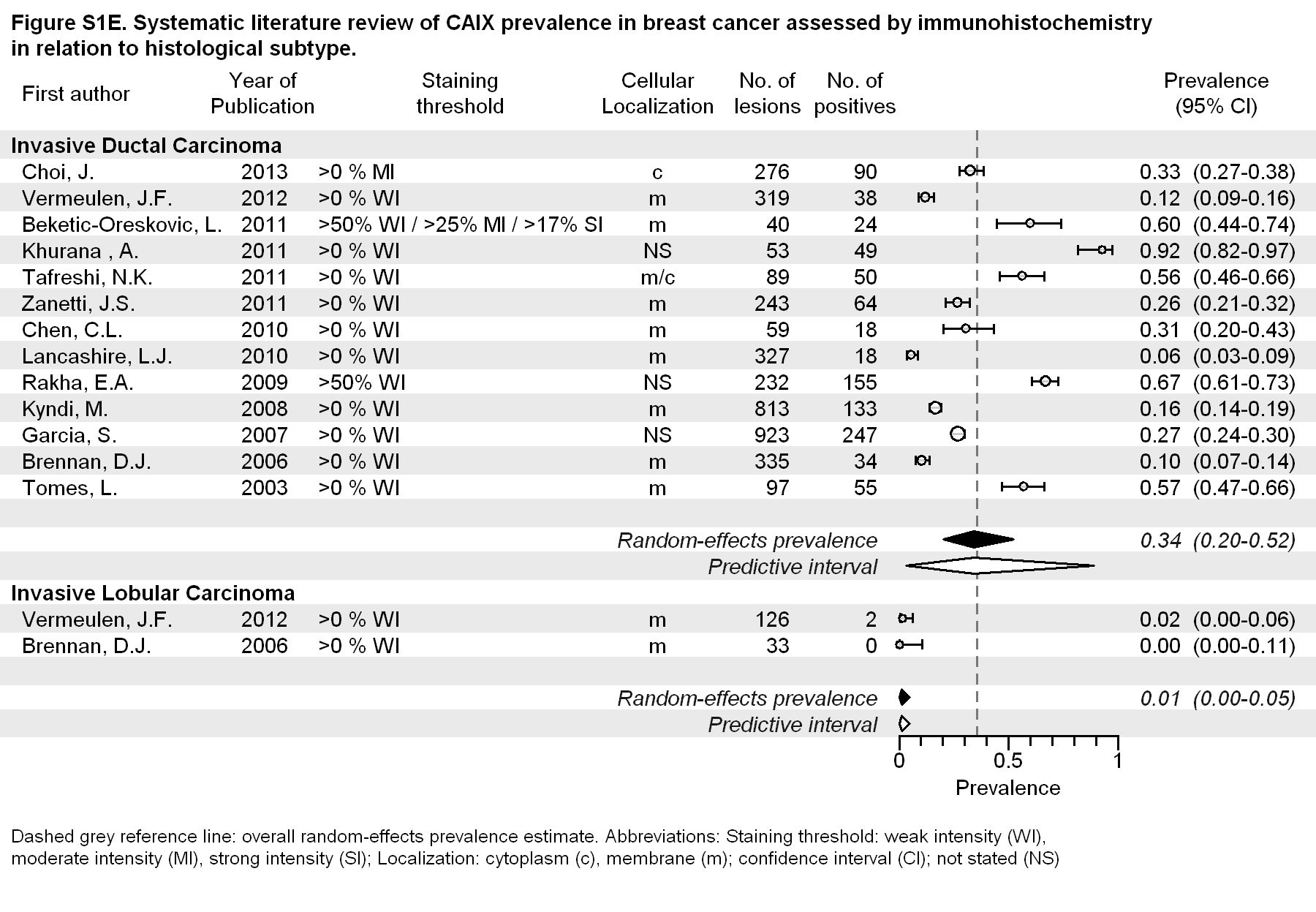

Supplement: Additional file 9: Figure S1E — CAIX - Histology. Systematic literature review of CAIX prevalence in breast cancer assessed by immunohistochemistry in relation to histological subtype. [file 1471-2407-13-538-S9.jpeg]

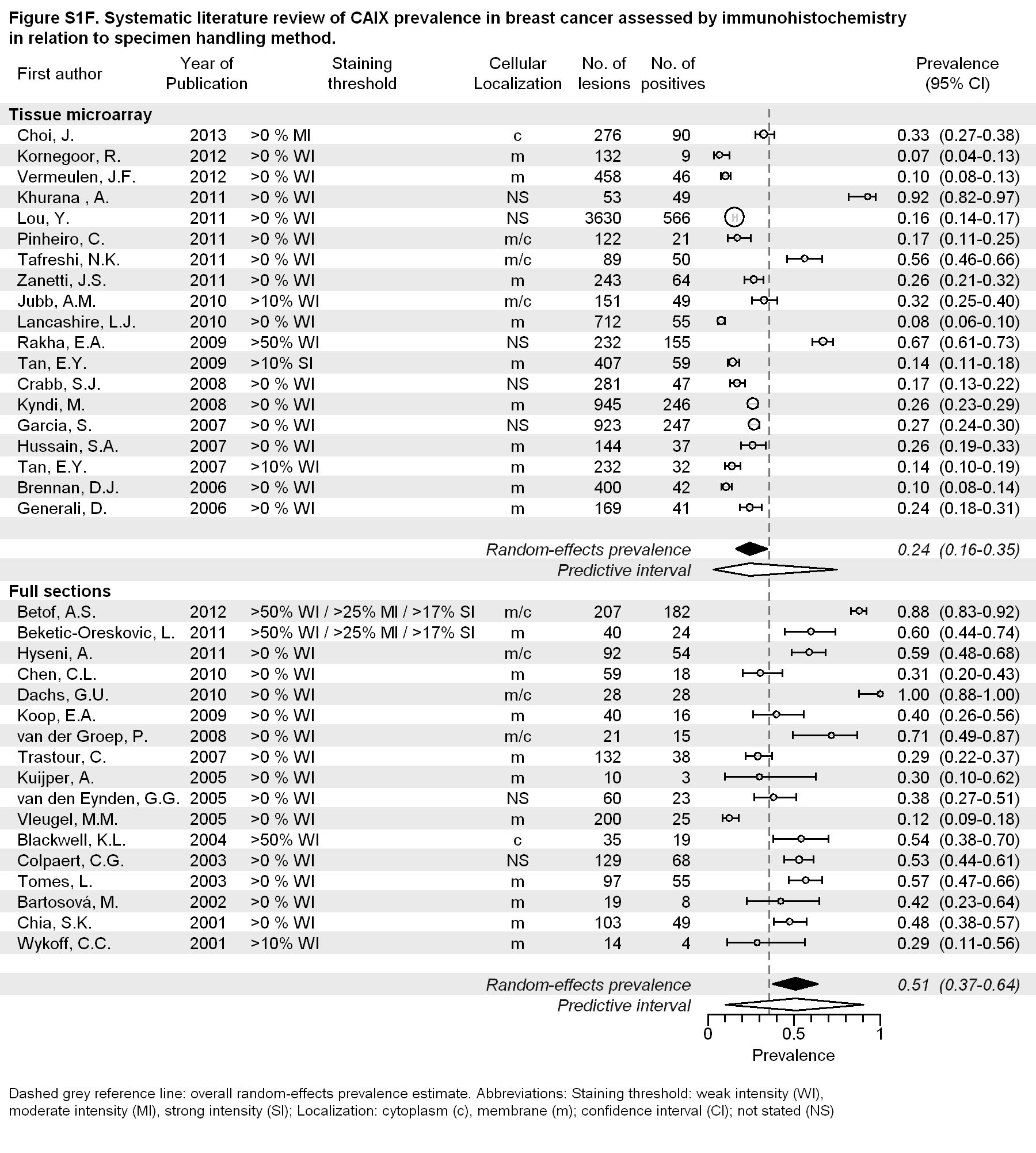

Supplement: Additional file 10: Figure S1F — CAIX - Specimen handling. Systematic literature review of CAIX prevalence in breast cancer assessed by immunohistochemistry in relation to specimen handling method. [file 1471-2407-13-538-S10.jpeg]

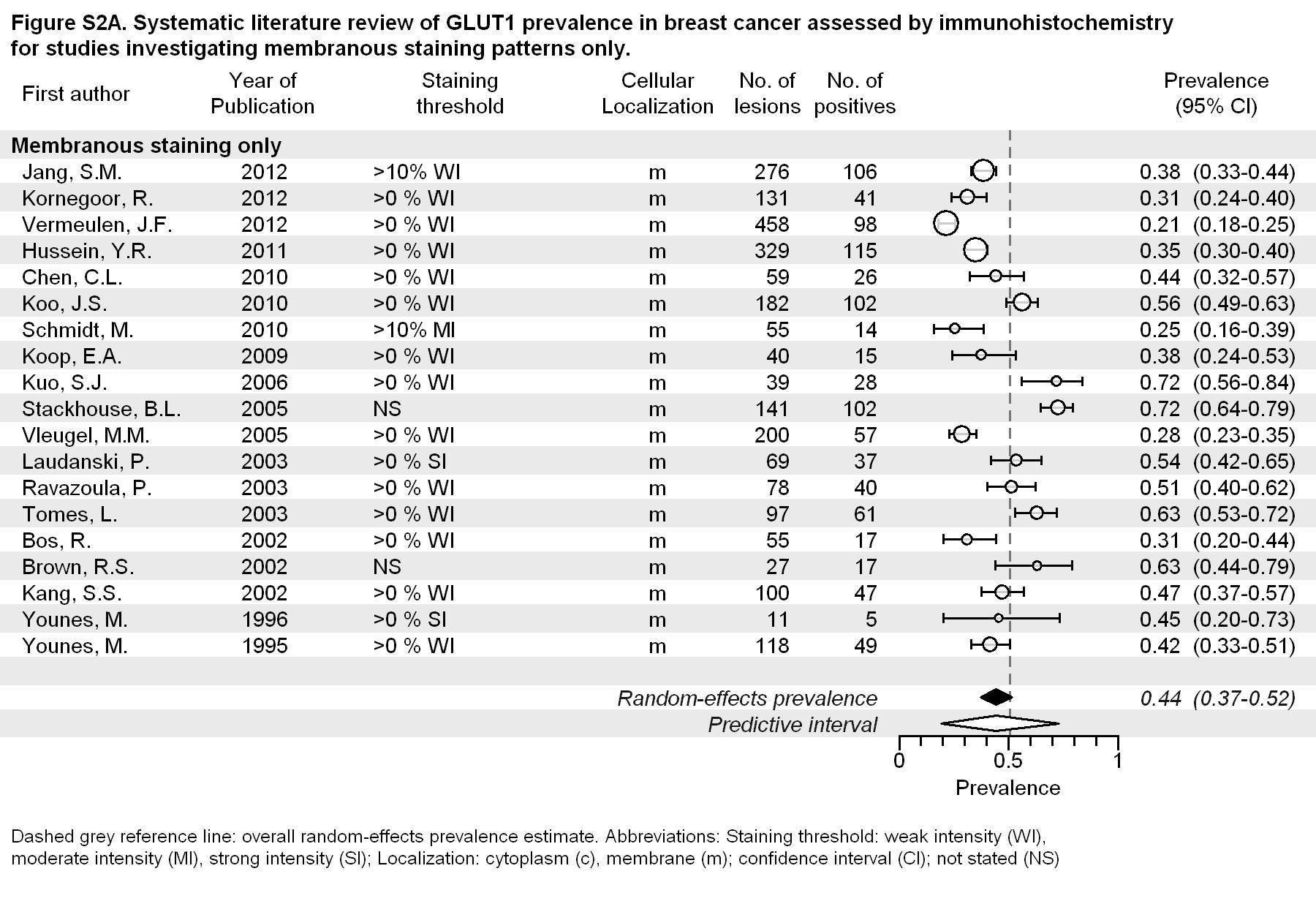

Supplement: Additional file 11: Figure S2A — GLUT1 - Membranous staining. Systematic literature review of GLUT1 prevalence in breast cancer assessed by immunohistochemistry for studies investigating membranous staining patterns only. [file 1471-2407-13-538-S11.jpeg]

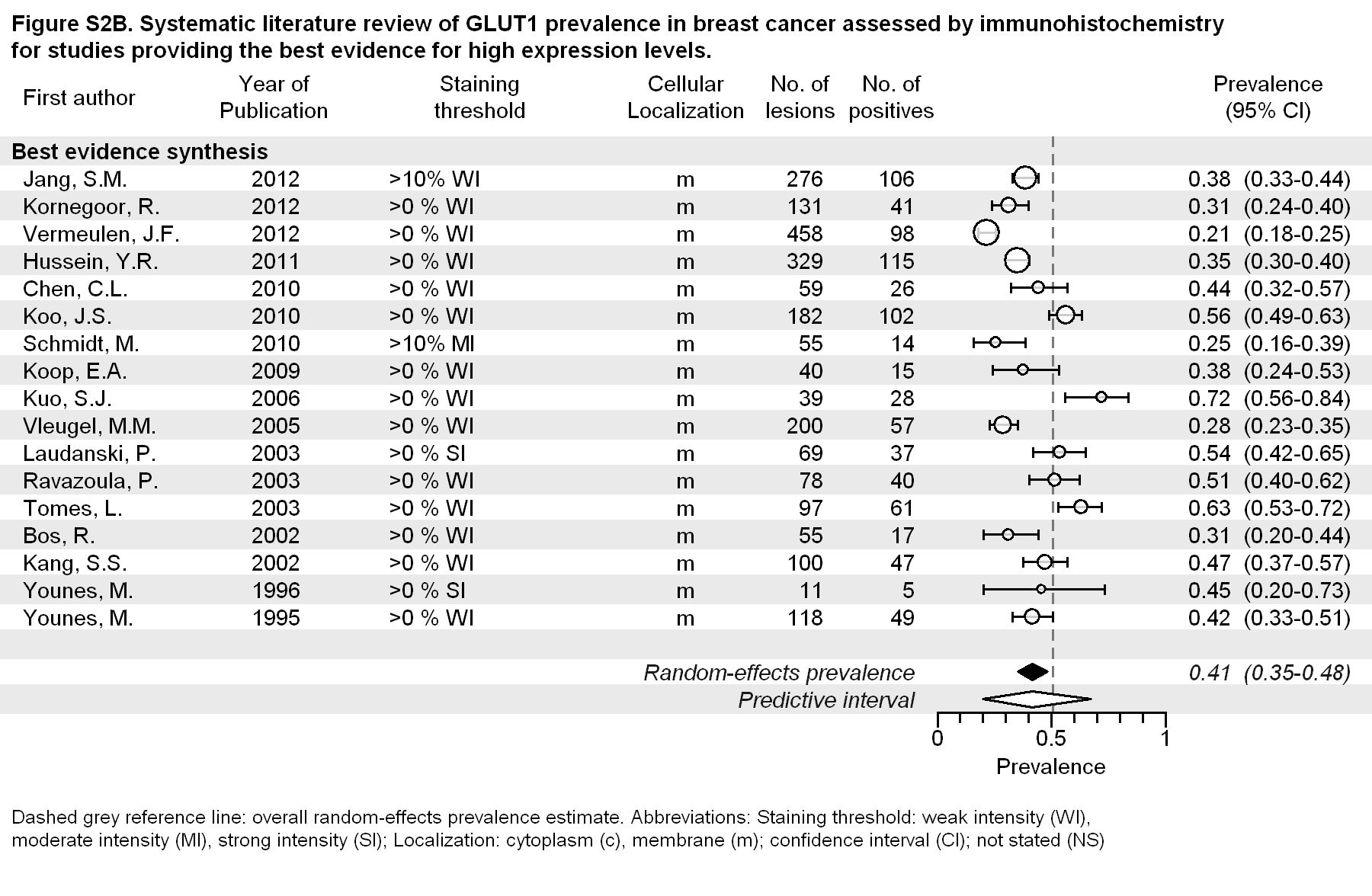

Supplement: Additional file 12: Figure S2B — GLUT1 - Best evidence studies. Systematic literature review of GLUT1 prevalence in breast cancer assessed by immunohistochemistry for studies providing the best evidence for high expression levels. [file 1471-2407-13-538-S12.jpeg]

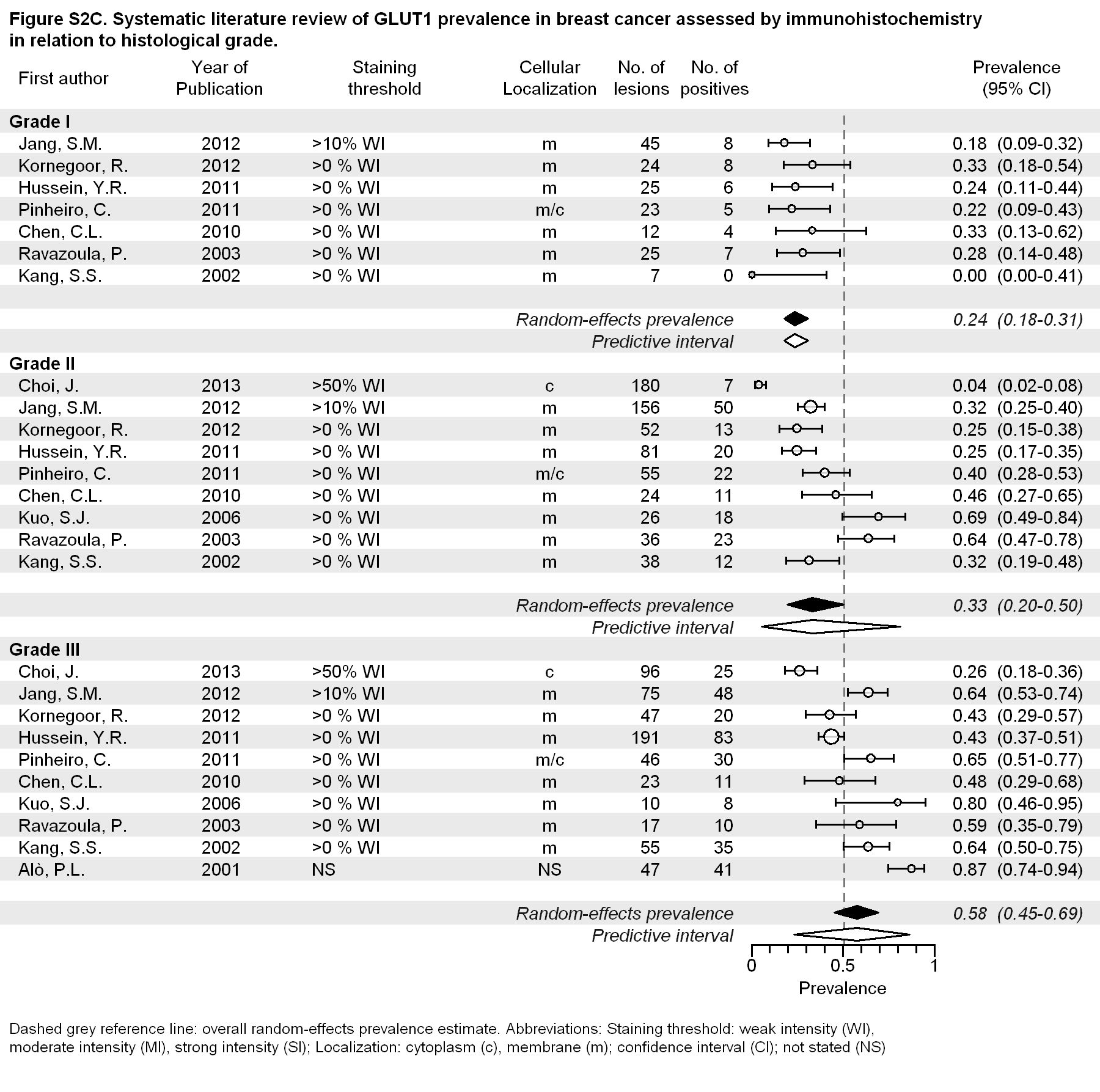

Supplement: Additional file 13: Figure S2C — GLUT1 - Histological grade. Systematic literature review of GLUT1 prevalence in breast cancer assessed by immunohistochemistry in relation to histological grade. [file 1471-2407-13-538-S13.jpeg]

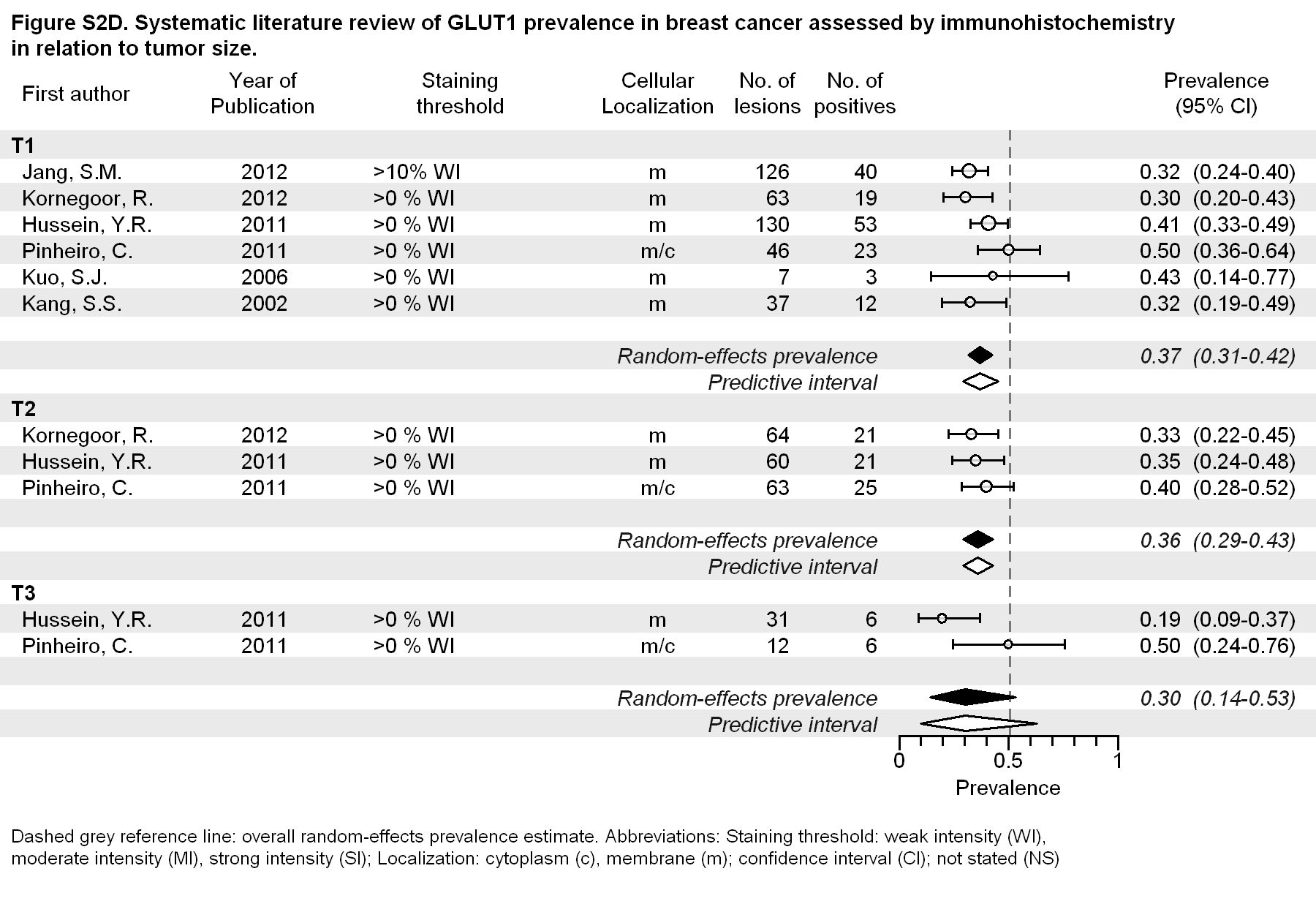

Supplement: Additional file 14: Figure S2D — GLUT1 - Tumor size. Systematic literature review of GLUT1 prevalence in breast cancer assessed by immunohistochemistry in relation to tumor size. [file 1471-2407-13-538-S14.jpeg]

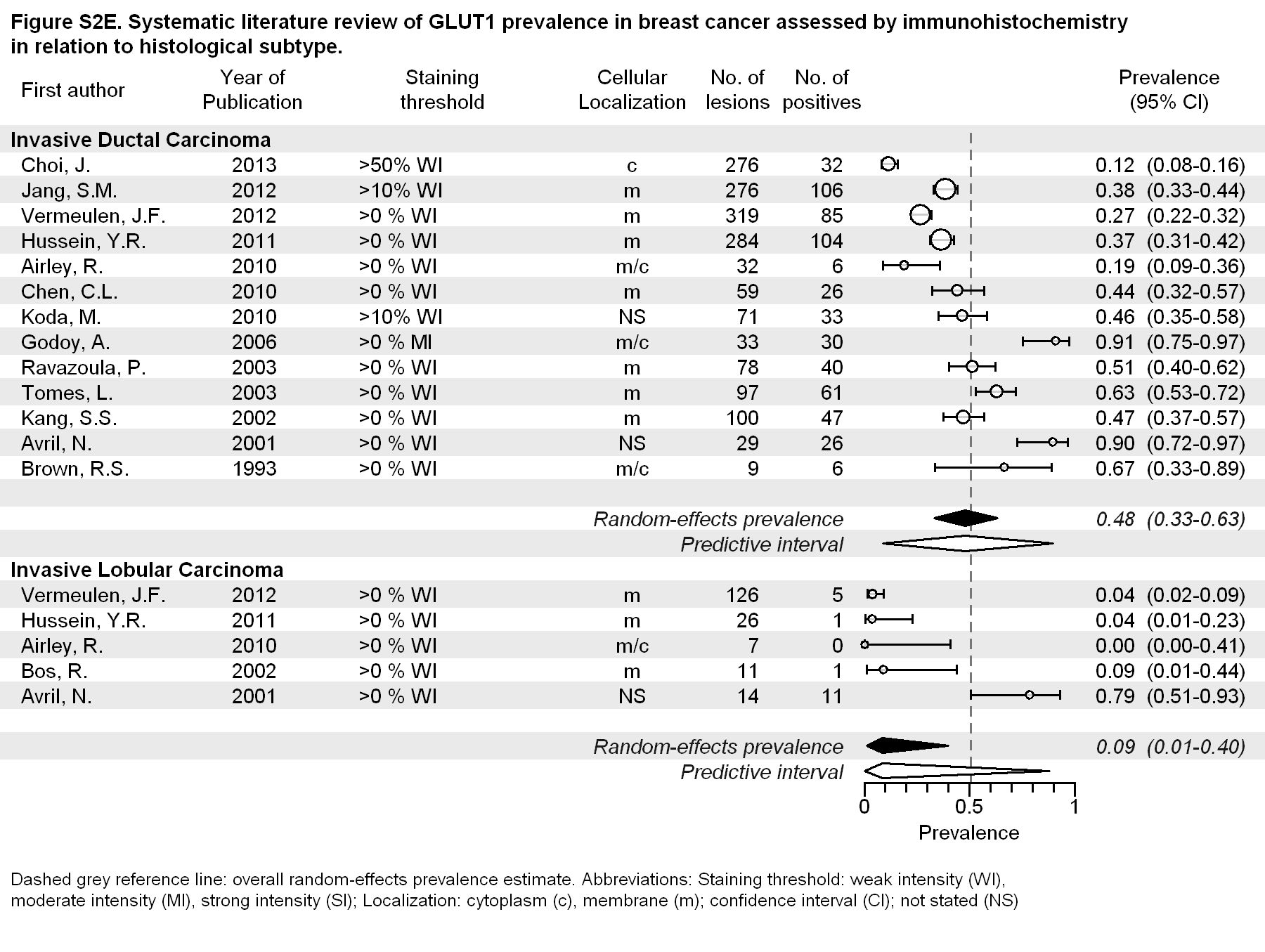

Supplement: Additional file 15: Figure S2E — GLUT1 - Histology. Systematic literature review of GLUT1 prevalence in breast cancer assessed by immunohistochemistry in relation to histological subtype. [file 1471-2407-13-538-S15.jpeg]

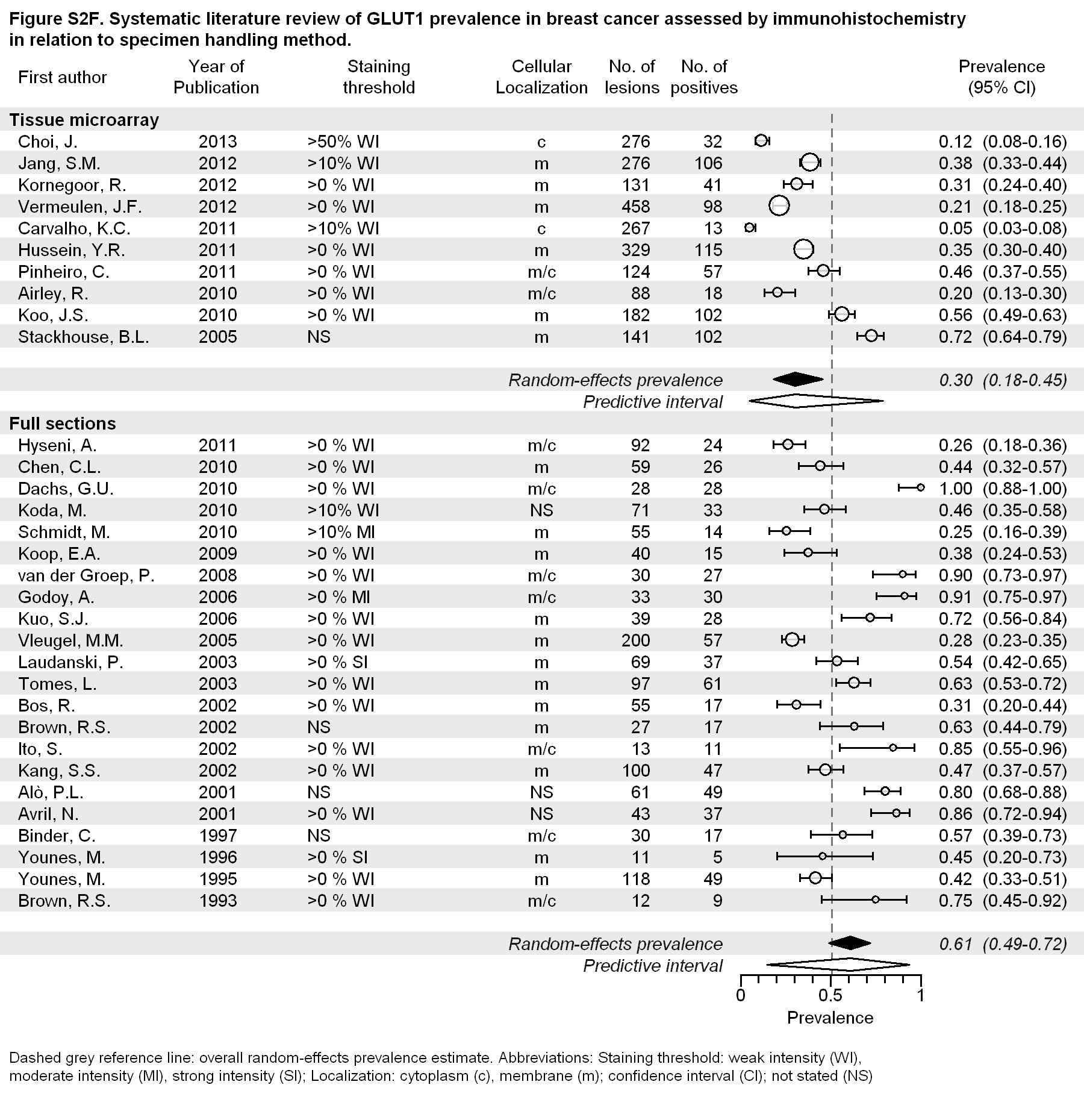

Supplement: Additional file 16: Figure S2F — GLUT1 - Specimen handling. Systematic literature review of GLUT1 prevalence in breast cancer assessed by immunohistochemistry in relation to specimen handling method. [file 1471-2407-13-538-S16.jpeg]

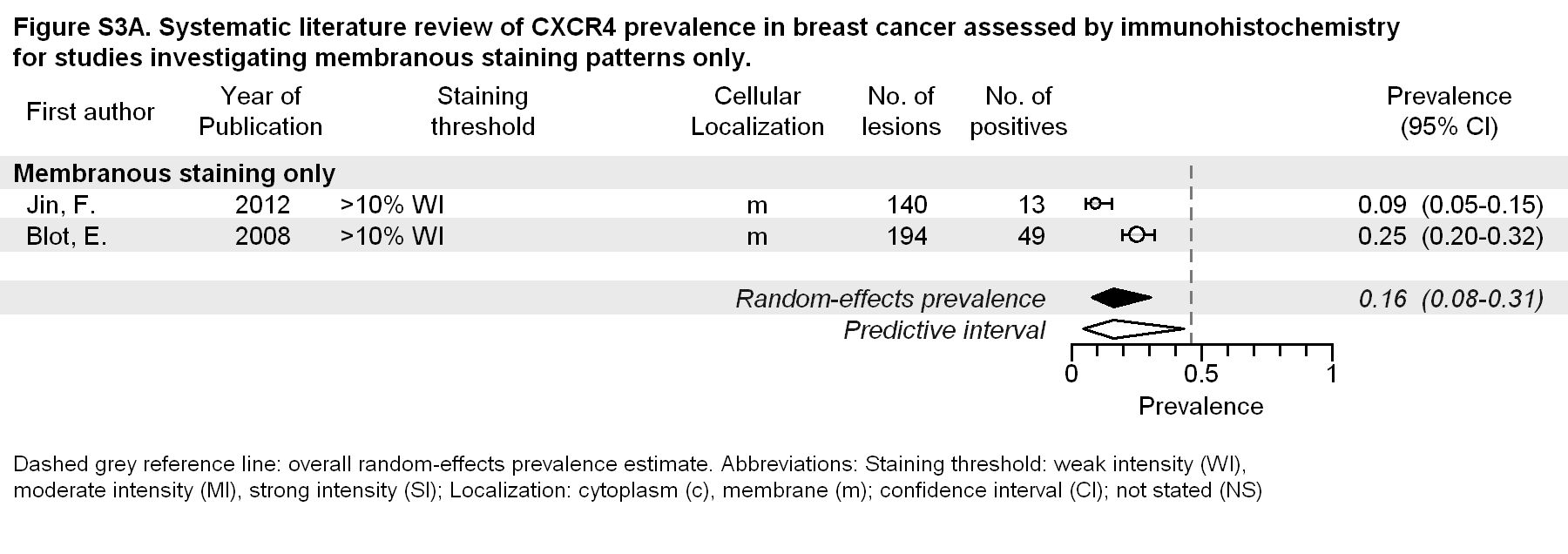

Supplement: Additional file 17: Figure S3A — CXCR4 - Membranous staining. Systematic literature review of CXCR4 prevalence in breast cancer assessed by immunohistochemistry for studies investigating membranous staining patterns only. [file 1471-2407-13-538-S17.jpeg]

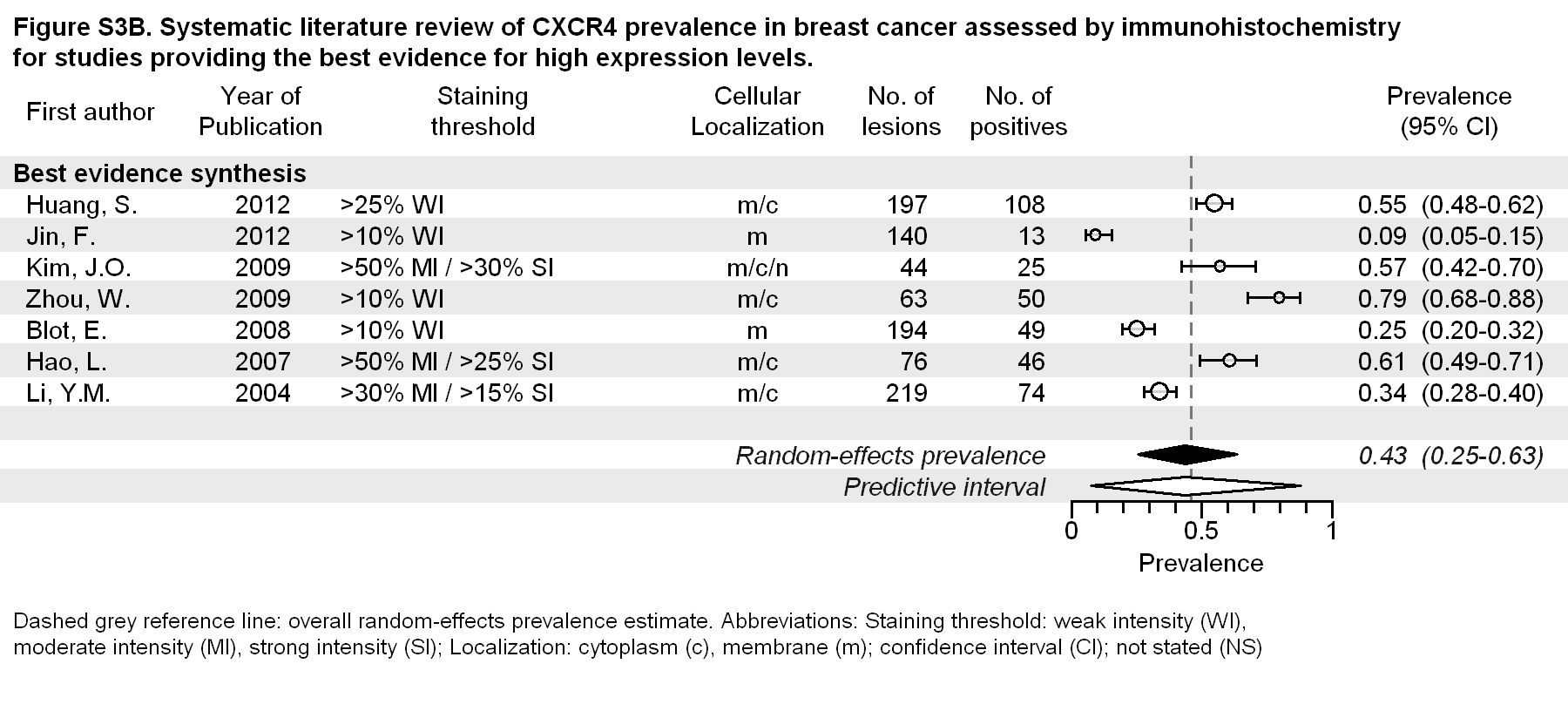

Supplement: Additional file 18: Figure S3B — CXCR4 - Best evidence studies. Systematic literature review of CXCR4 prevalence in breast cancer assessed by immunohistochemistry for studies providing the best evidence for high expression levels. [file 1471-2407-13-538-S18.jpeg]

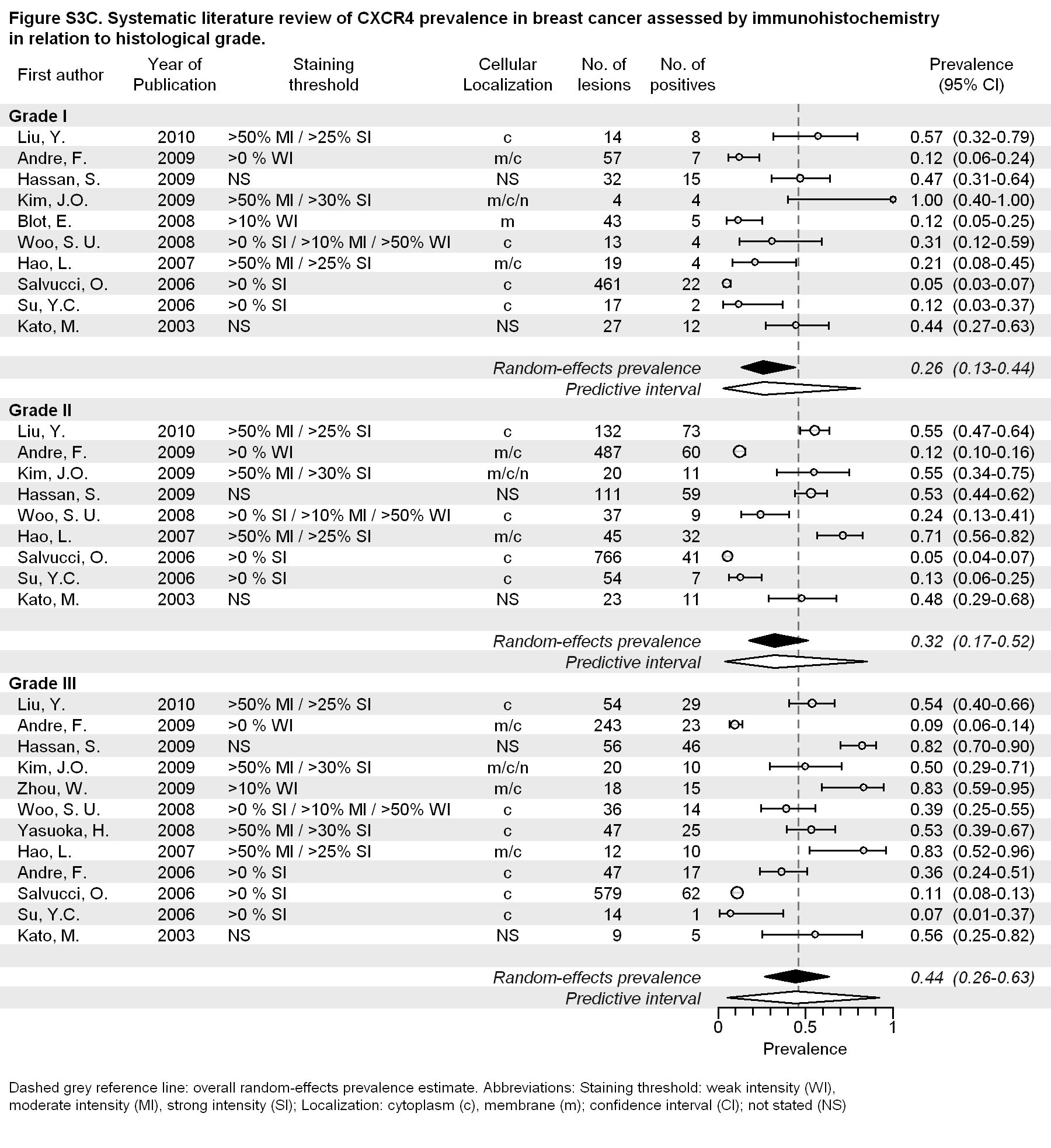

Supplement: Additional file 19: Figure S3C — CXCR4 - Histological grade. Systematic literature review of CXCR4 prevalence in breast cancer assessed by immunohistochemistry in relation to histological grade. [file 1471-2407-13-538-S19.jpeg]

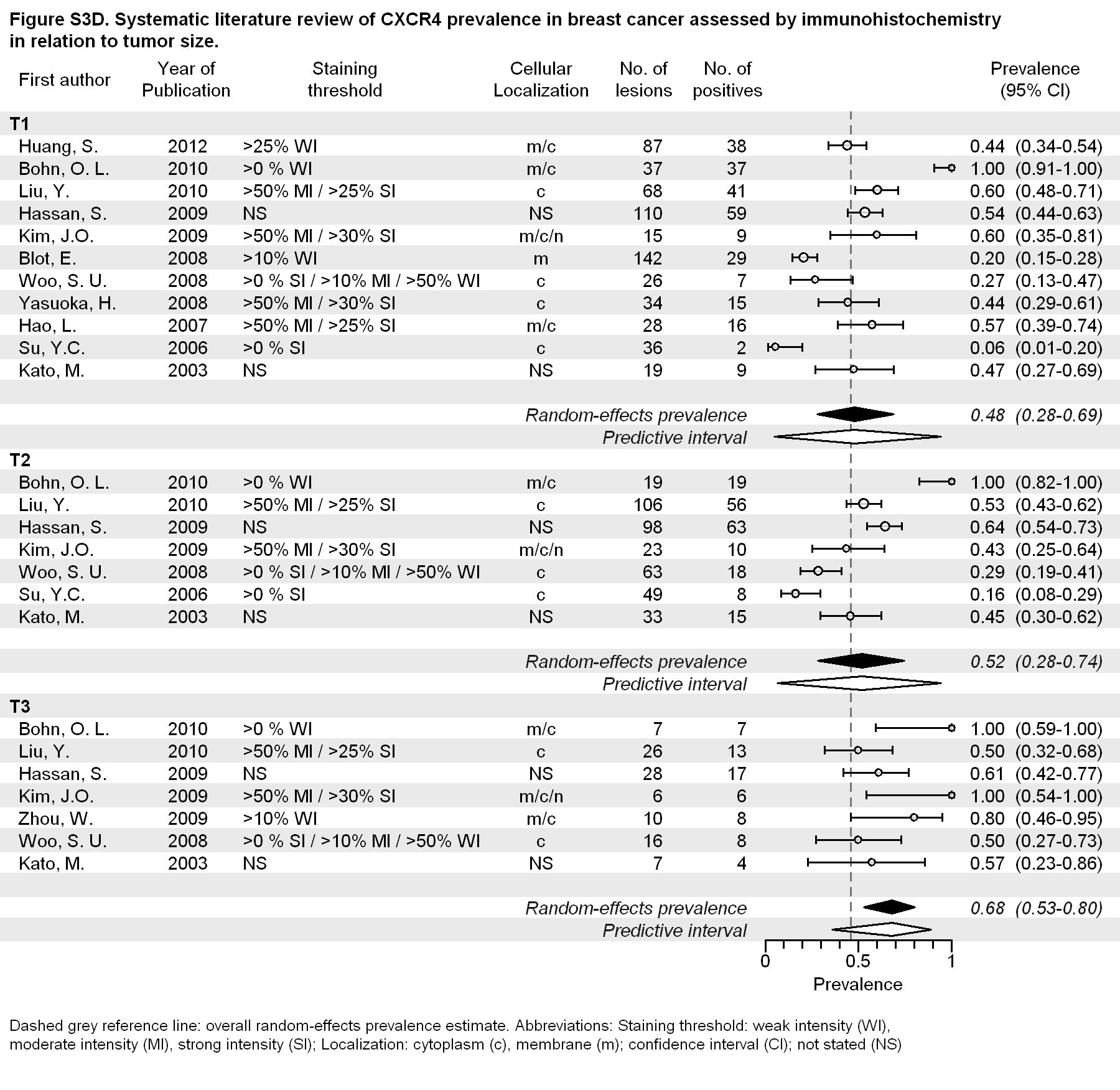

Supplement: Additional file 20: Figure S3D — CXCR4 - Tumor size. Systematic literature review of CXCR4 prevalence in breast cancer assessed by immunohistochemistry in relation to tumor size. [file 1471-2407-13-538-S20.jpeg]

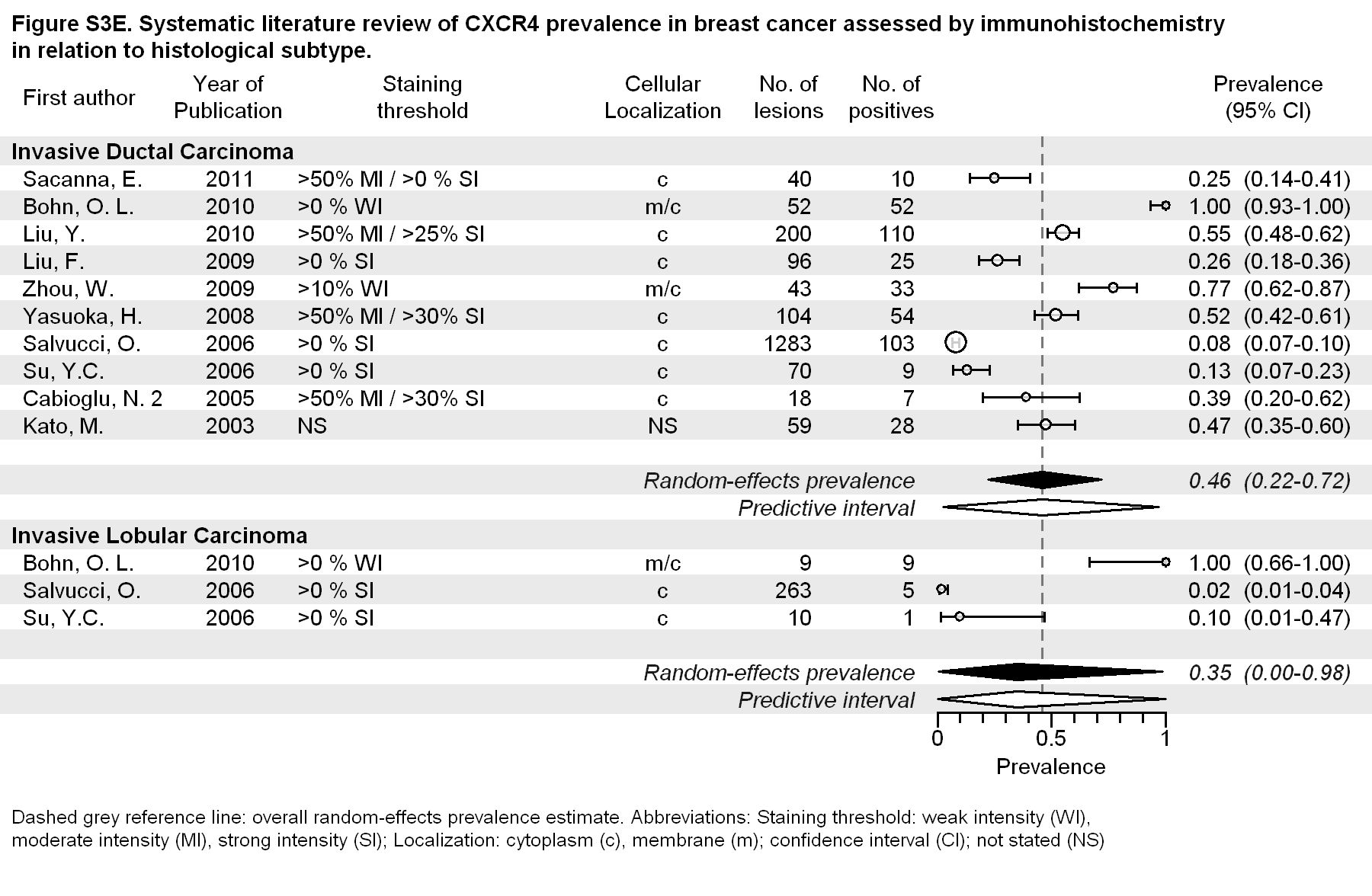

Supplement: Additional file 21: Figure S3E — CXCR4 - Histology. Systematic literature review of CXCR4 prevalence in breast cancer assessed by immunohistochemistry in relation to histological subtype. [file 1471-2407-13-538-S21.jpeg]

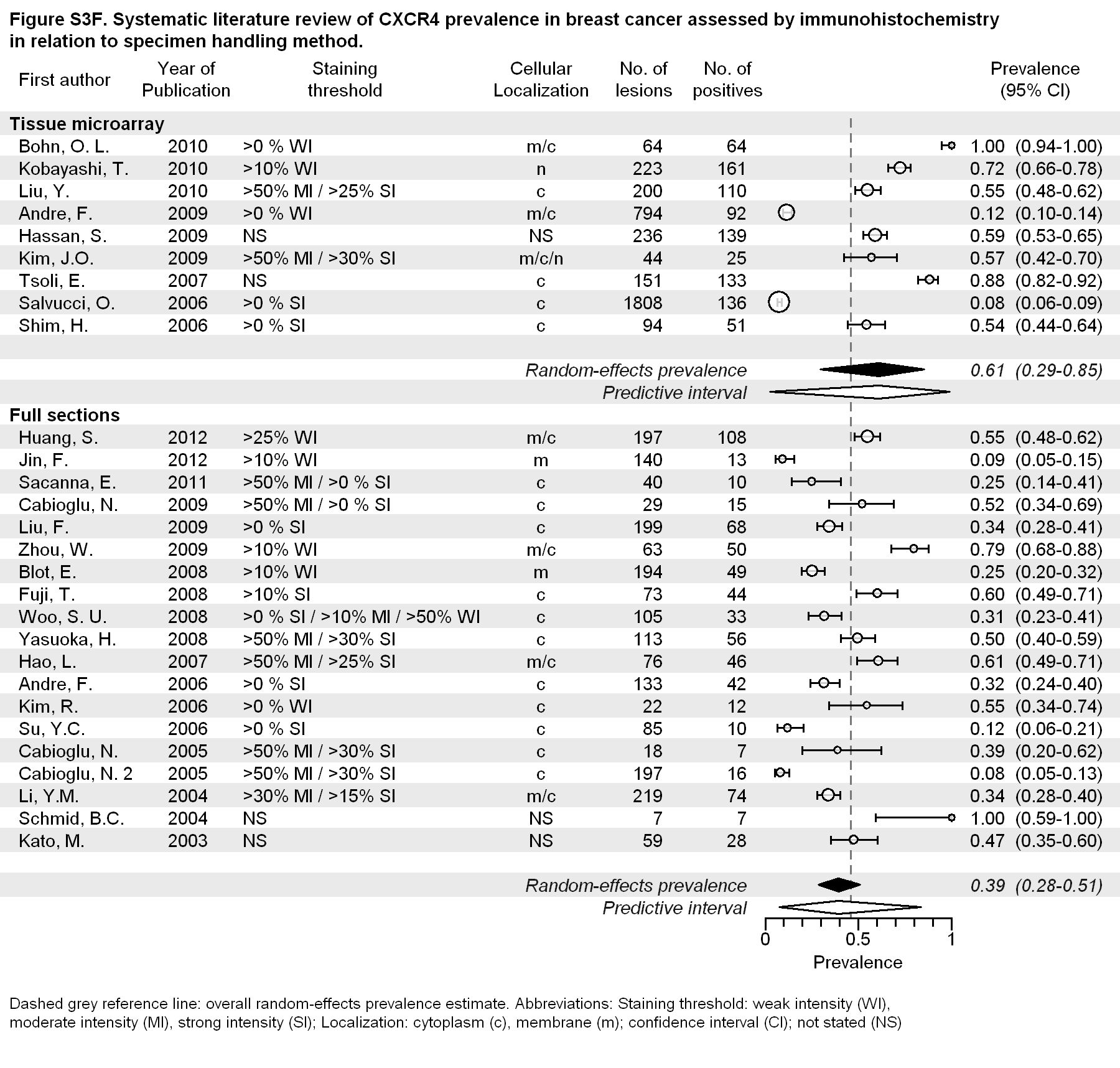

Supplement: Additional file 22: Figure S3F — CXCR4 - Specimen handling. Systematic literature review of CXCR4 prevalence in breast cancer assessed by immunohistochemistry in relation to specimen handling method. [file 1471-2407-13-538-S22.jpeg]

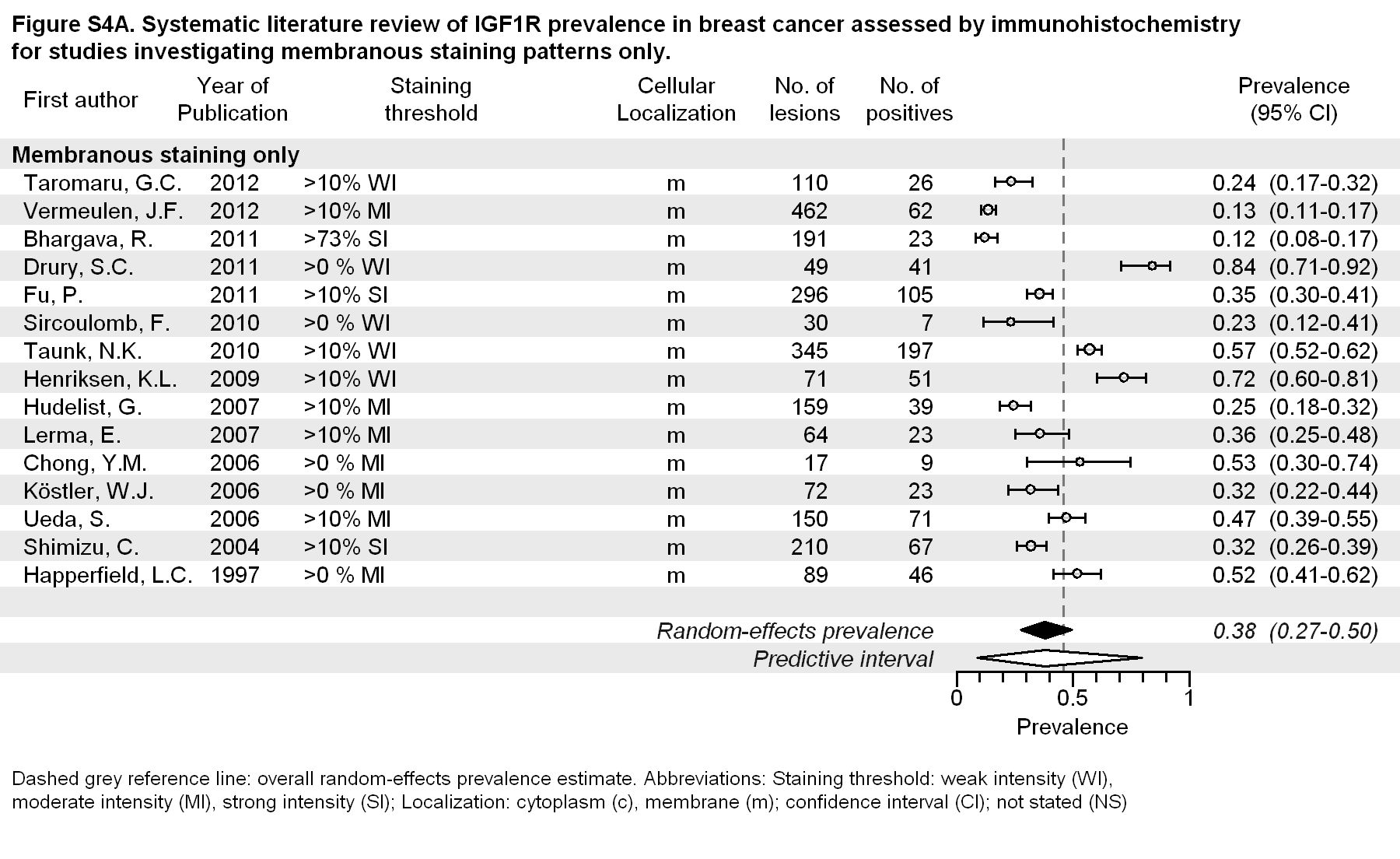

Supplement: Additional file 23: Figure S4A — IGF1R - Membranous staining. Systematic literature review of IGF1R prevalence in breast cancer assessed by immunohistochemistry for studies investigating membranous staining patterns only. [file 1471-2407-13-538-S23.jpeg]

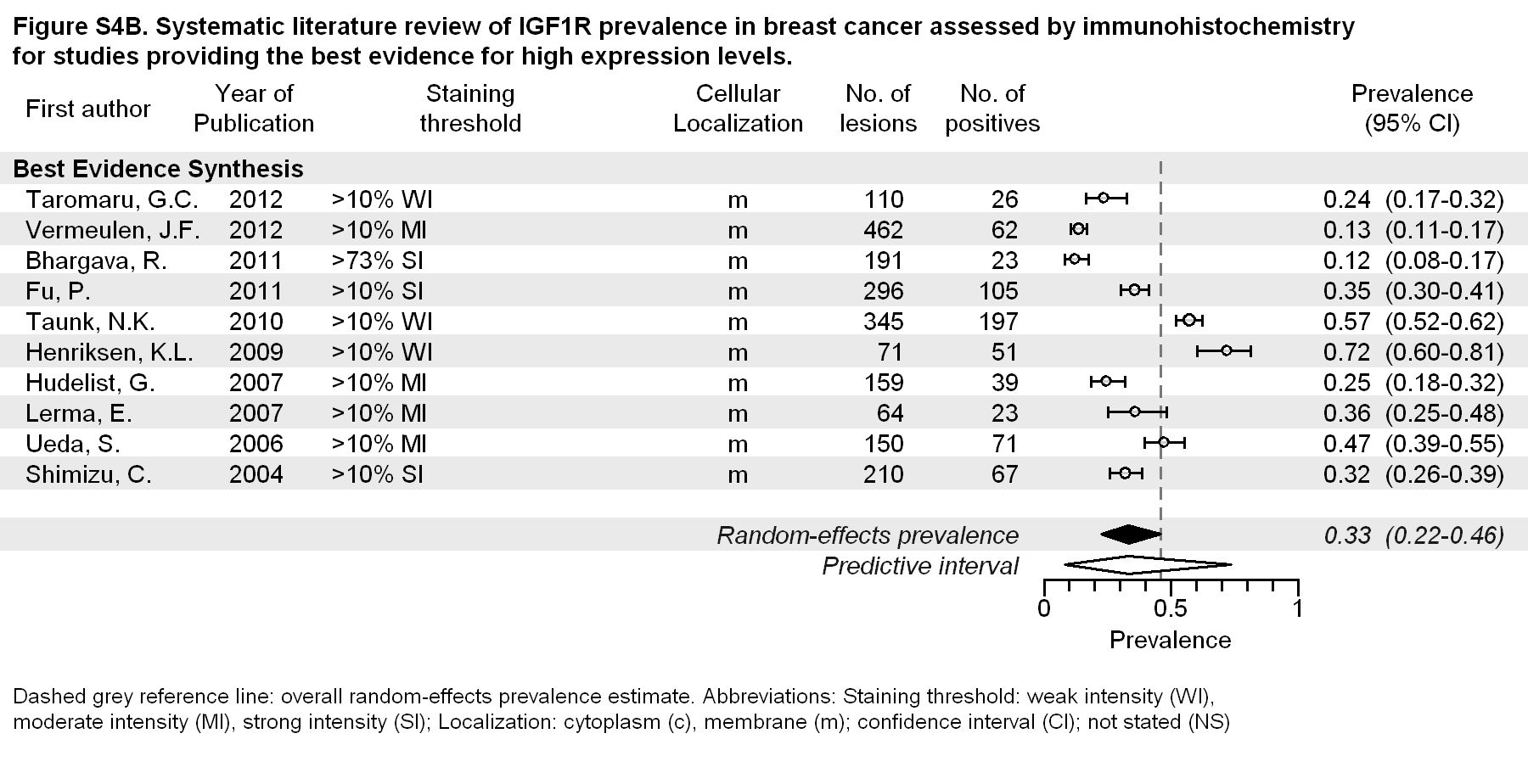

Supplement: Additional file 24: Figure S4B — IGF1R - Best evidence studies. Systematic literature review of IGF1R prevalence in breast cancer assessed by immunohistochemistry for studies providing the best evidence for high expression levels. [file 1471-2407-13-538-S24.jpeg]

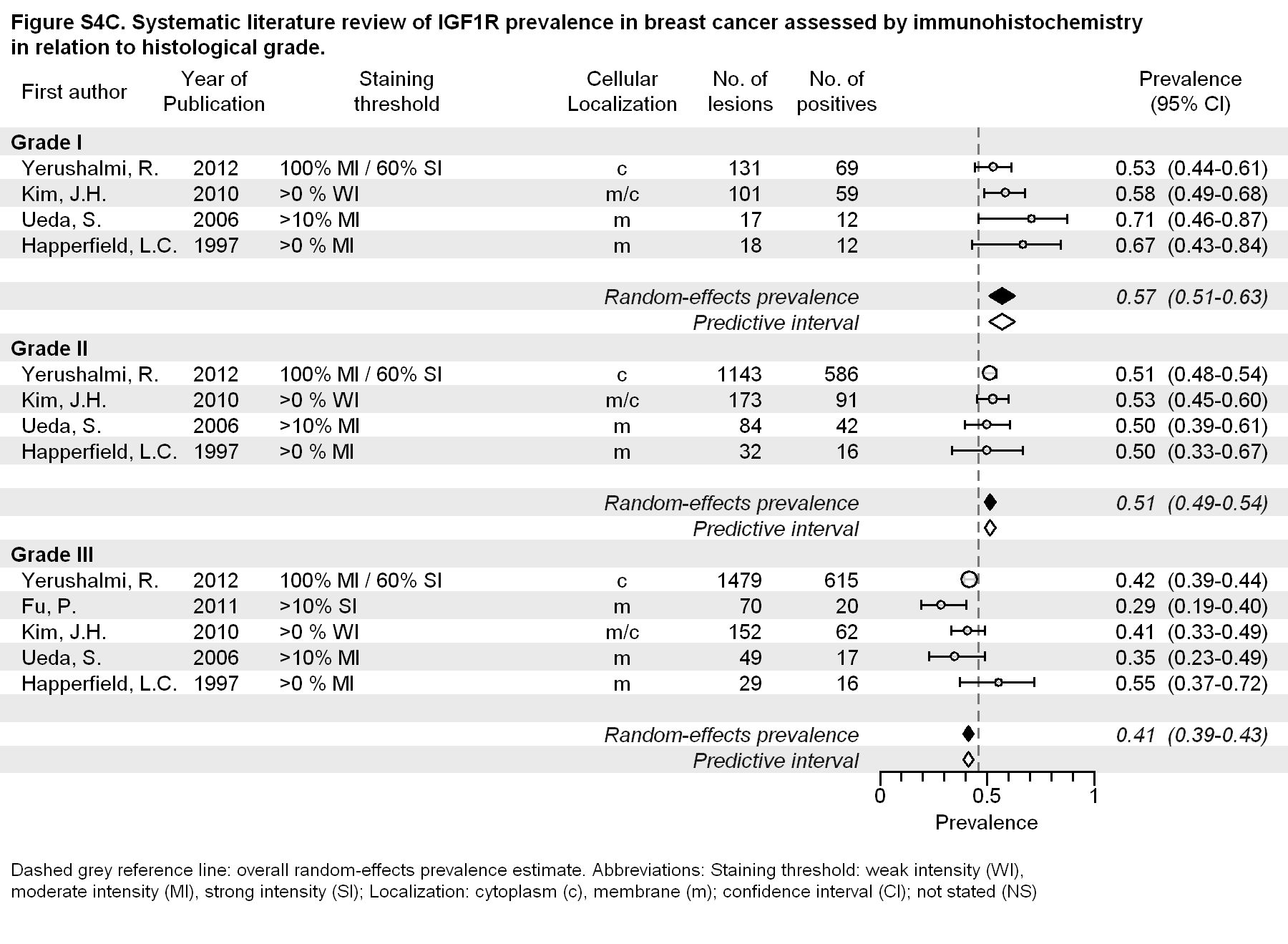

Supplement: Additional file 25: Figure S4C — IGF1R - Histological grade. Systematic literature review of IGF1R prevalence in breast cancer assessed by immunohistochemistry in relation to histological grade. [file 1471-2407-13-538-S25.jpeg]

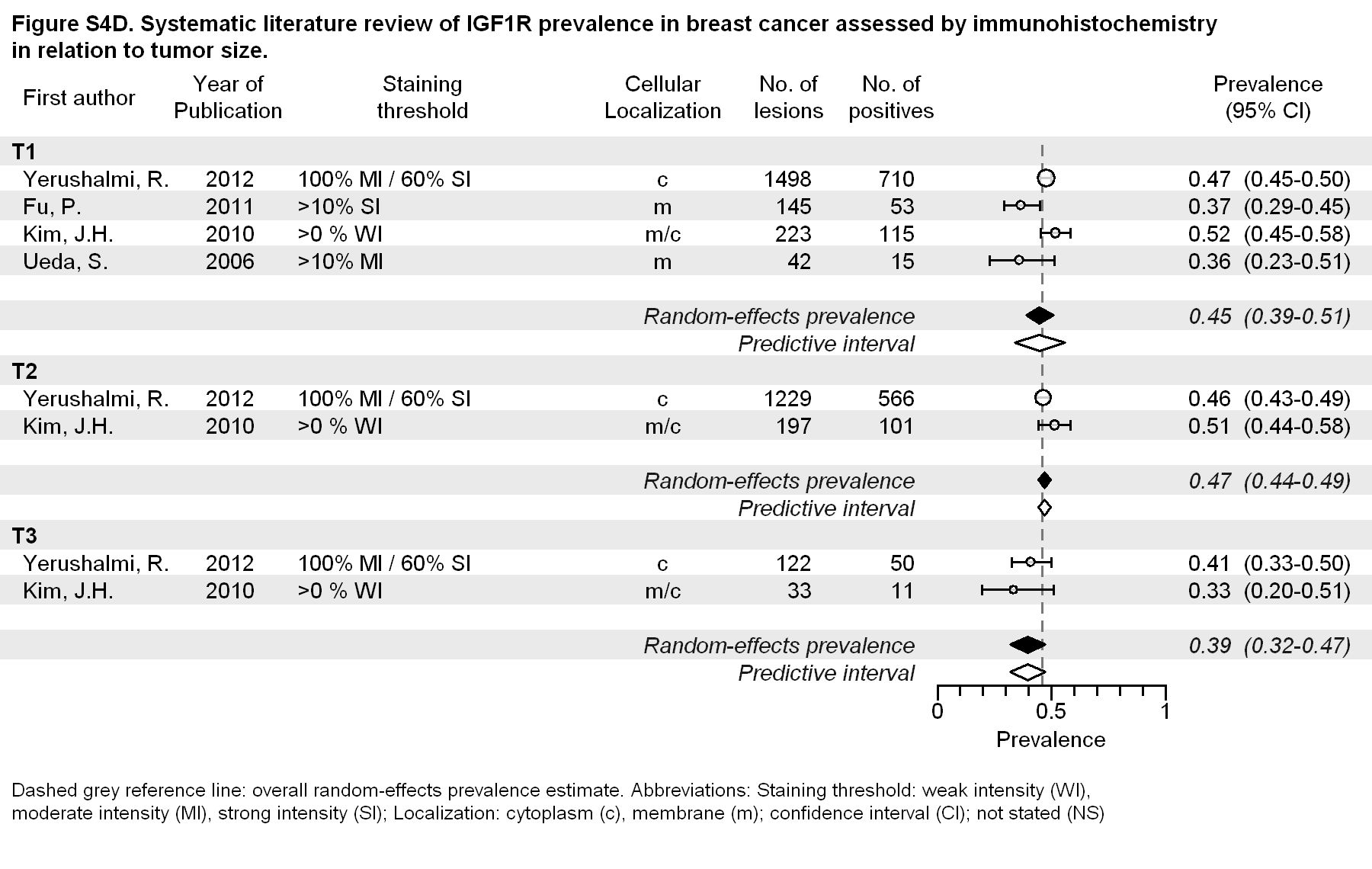

Supplement: Additional file 26: Figure S4D — IGF1R - Tumor size. Systematic literature review of IGF1R prevalence in breast cancer assessed by immunohistochemistry in relation to tumor size. [file 1471-2407-13-538-S26.jpeg]

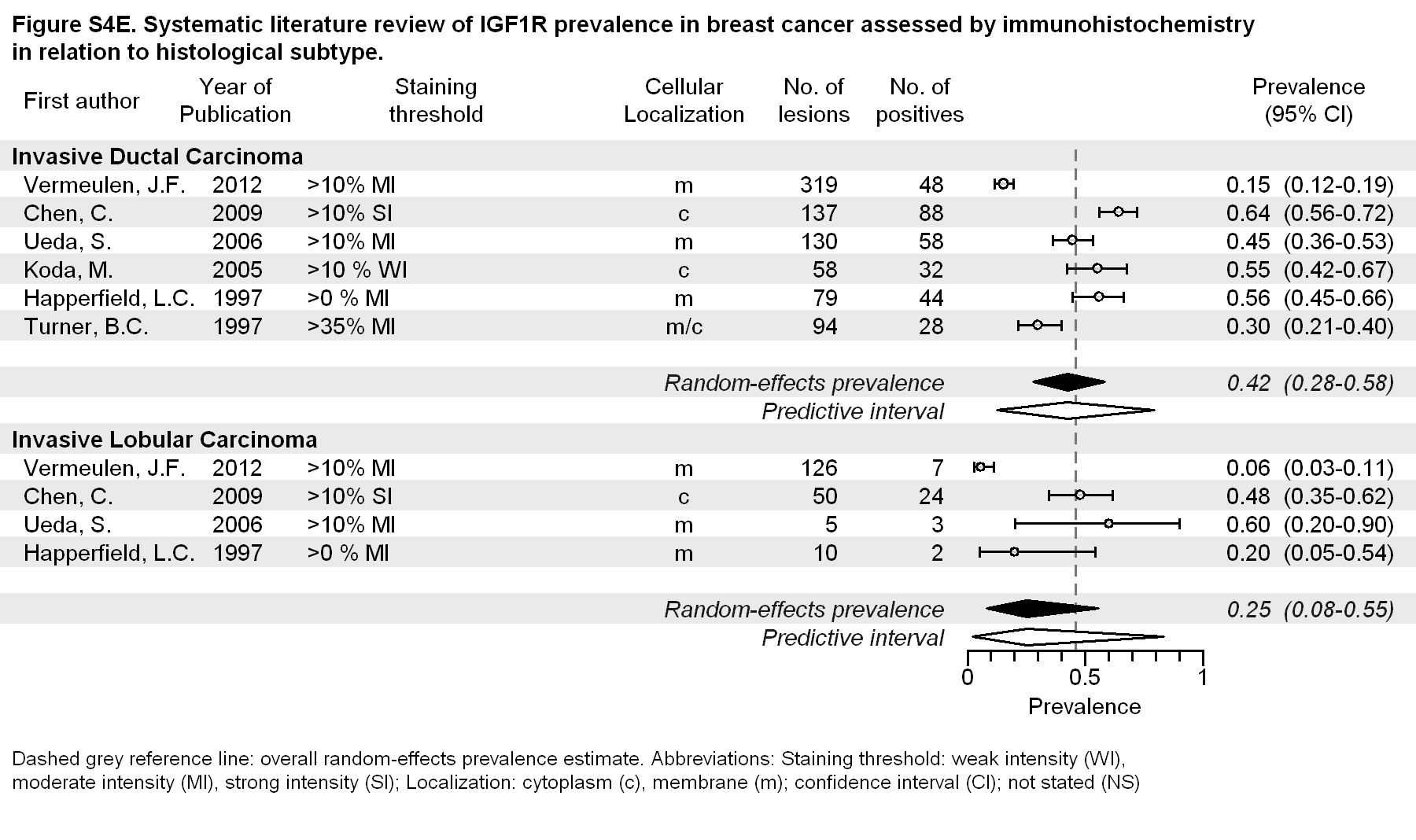

Supplement: Additional file 27: Figure S4E — IGF1R - Histology. Systematic literature review of IGF1R prevalence in breast cancer assessed by immunohistochemistry in relation to histological subtype. [file 1471-2407-13-538-S27.jpeg]

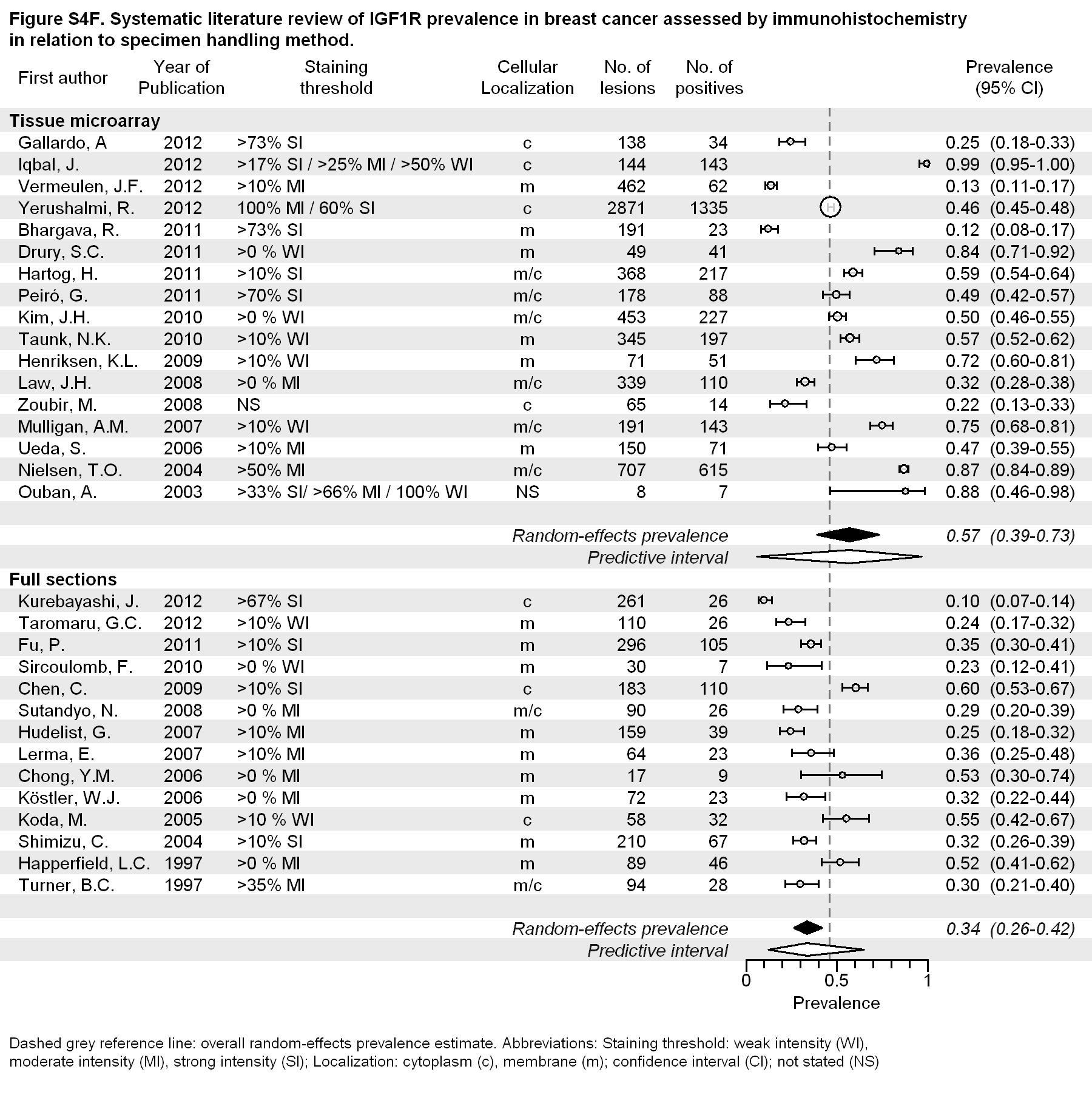

Supplement: Additional file 28: Figure S4F — IGF1R - Specimen handling. Systematic literature review of IGF1R prevalence in breast cancer assessed by immunohistochemistry in relation to specimen handling method. [file 1471-2407-13-538-S28.jpeg]
